# Iron Oxide Nanoparticle-Based T_1_ Contrast Agents for Magnetic Resonance Imaging: A Review

**DOI:** 10.3390/nano15010033

**Published:** 2024-12-28

**Authors:** Dongmei Zhang, Jing Zhang, Xianglin Bian, Pei Zhang, Weihua Wu, Xudong Zuo

**Affiliations:** 1 School of Mathematics and Physics, Jiangsu University of Technology, Changzhou 213100, China; zhdm@jsut.edu.cn (D.Z.);; 2 The Jiangsu Key Laboratory of Clean Energy Storage and Conversion, Jiangsu University of Technology, Changzhou 213100, China; 3 Jiangsu Key Laboratory for Biomaterials and Devices, School of Biological Science and Medical Engineering, Southeast University, Nanjing 210096, China

**Keywords:** iron oxide nanoparticles, magnetic resonance imaging, T_1_ contrast agents

## Abstract

This review highlights recent progress in utilizing iron oxide nanoparticles (IONPs) as a safer alternative to gadolinium-based contrast agents (GBCAs) for magnetic resonance imaging (MRI). It consolidates findings from multiple studies, discussing current T_1_ contrast agents (CAs), the synthesis techniques for IONPs, the theoretical principles for designing IONP-based MRI CAs, and the key factors that impact their T_1_ contrast efficacy, such as nanoparticle size, morphology, surface modifications, valence states, and oxygen vacancies. Furthermore, we summarize current strategies to achieve IONP-based responsive CAs, including self-assembly/disassembly and distance adjustment. This review also evaluates the biocompatibility, organ accumulation, and clearance pathways of IONPs for clinical applications. Finally, the challenges associated with the clinical translation of IONP-based T_1_ CAs are included.

## 1. Introduction

Magnetic resonance imaging (MRI) is a widely utilized clinical diagnostic tool known for its excellent soft tissue contrast, absence of ionizing radiation, and capability of deep tissue imaging [1,2,3]. These advantages make MRI a preferred method for detecting serious illnesses like cancer and monitoring various diseases. However, to enhance sensitivity and accurately differentiate diseased tissue from healthy tissue, -especially in the early stages, the use of contrast agents (CAs) becomes essential [4]. These agents improve imaging contrast by shortening the longitudinal (T_1_) and transverse (T_2_) relaxation times of water protons, resulting in higher image quality and expanding non-invasive visualization possibilities [3], thereby enhancing clinical utility and patient care. The effectiveness of these agents is evaluated through longitudinal and transverse relaxivity parameters, r_1_ and r_2_, which represent T_1_ and T_2_ relaxation rate enhancements per mM concentration of agents (units mM^−1^s^−1^), indicating their potential for bright and dark signal enhancement, respectively.

T_2_ CAs, such as superparamagnetic nanoparticles, have high susceptibility, generating long-range magnetic fields that disrupt neighboring tissues and cause background distortion, commonly referred to as the “blooming effect” [5,6]. This can lead to misidentification of T_2_ CAs’ locations and confusion with other low-intensity areas due to bleeding, calcification, or metal deposits [7]. In contrast, T_1_ CAs, composed of paramagnetic ions, do not interfere with magnetic uniformity or anatomical backgrounds, enabling clearer detection. Because of these drawbacks associated with T_2_ CAs, T_1_ CAs, particularly gadolinium (Gd) complexes, are more frequently adopted in clinical practice [8].

Nevertheless, Gd-based complexes pose significant health risks, particularly for patients with kidney and liver dysfunctions, who struggle to eliminate heavy metal complexes efficiently [9]. This can result in severe complications, including nephrogenic systemic fibrosis syndrome [10,11], even in individuals without pre-existing conditions. Additionally, the short circulation lifetimes of gadolinium-based contrast agents (GBCAs) often require multiple administrations, further increasing associated risks [12]. Consequently, there is a strong demand for T_1_ CAs that demonstrate good biocompatibility.

Iron oxide nanoparticles (IONPs) have emerged as promising candidates in the biomedical field due to their favorable biocompatibility [13,14,15,16]. They display extended blood half-lives, low toxicity, and flexible surface chemistry, allowing for efficient clearance from the body through natural degradation into hemoglobin. The compatibility of iron enhances the appeal of IONPs as potential T_1_ CAs [3,5,17,18,19], offering a combination of efficacy and safety for clinical applications.

Although various IONP systems have been investigated as potential T_1_ CAs, the synthesis techniques used differ widely, and the mechanisms affecting their efficacy are still not well understood. This review focuses on essential elements of employing IONPs as T1-MRI CAs, encompassing synthesis methods, basic principles of MRI CAs, key factors that influence T_1_-MRI performance, and approaches for designing responsive CAs. Additionally, we discuss the challenges associated with developing these CAs, suggest possible solutions, and outline considerations to help translate research findings into clinical applications.

## 2. Current T_1_-MRI CAs

### 2.1. Gd-Based CAs

The T_1_-MRI CAs market has long been dominated by commercial Gd-chelates such as Omniscan, Dotarem, OptiMARK, Magnevist, Eovist, Gadovist, Multihance, and ProHance [19]. These agents rapidly accumulate in diseased areas and are quickly excreted by the kidneys. However, they have drawbacks like potential nephrotoxicity, brain deposition, and lack of tissue or cell specificity [10,20,21].

Nanoparticles, on the other hand, offer extended blood circulation time and better biocompatibility. They can be functionalized with various chemicals, targeting ligands, and fluorescent dyes for multimodal imaging and therapy. Recent advancements include Gd_2_O_3_, NaGdF, GdF, and Gd-doped nanoparticles, which have shown promise as T_1_ contrast agents [3].

Li et al. introduced an MRA imaging method using long-circulating BSA-Gd_2_O_3_ nanoparticles for diagnosing carotid artery stenosis [22]. These nanoparticles, synthesized through biomineralization, provided sustained enhancement for 2 h at half the clinical dose, surpassing Gd-DTPA. They enabled clear visualization of vessels as small as 0.3 mm and precise diagnosis of stenosis severity and location in rats, facilitating theranostics. Jiang et al. designed ultra-small NaGdF4 particles coated with a hydrophilic block copolymer, enabling clear imaging of tiny blood vessels (0.17 mm diameter, 2-hour window) at low magnetic field strengths (1.0 T) [23]. This approach reduces NSF risks compared to Gd-DTPA and shows potential for accurate diagnosis of vascular diseases.

While Gd-based nanoparticles hold significant clinical potential, critics often highlight their challenges, such as poor water solubility, risk of Gd leakage, and uncertain kidney toxicity.

### 2.2. Mn-Based CAs

Mn^2+^ ions excel as T_1_ CAs due to their five unpaired electrons, which extend electronic relaxation and amplify T_1_ signals through dipolar interactions with protons. A commercially available MRI contrast agent for the hepatobiliary system is mangafodipir trisodium (Mn-DPDP), which employs DPDP as a biological ligand targeted by the pyridoxal phosphate (PLP) membrane transport system [24]. Mn-DPDP demonstrates high thermodynamic stability, with a lethal dose approximately 18 times higher than MnCl_2_, and partial renal excretion [25]. This has driven the development of advanced Mn-based chelates with enhanced stability and rapid in vivo clearance.

Moreover, manganese oxide nanoparticles (such as MnO, Mn_3_O_4_, and MnO_2_) have garnered significant attention as responsive MRI contrast agents for GSH or pH. Their easily alterable valence states facilitate the release of manganese in acidic environments, showing promise for responsive imaging. Chen et al. created hybrid mesoporous composite nanocapsules (HMCNs) with manganese oxide nanoparticles for pH-responsive T1-weighted MRI in cancer theranostics [26]. These HMCNs effectively detect tumor acidic microenvironments and show better relaxation properties than commercial Gd-based contrast agents. Moreover, HMCNs act as ultrasound contrast agents and enable intracellular delivery of anticancer drugs, aiding in overcoming multidrug resistance (MDR) and improving therapeutic effectiveness.

Yuan et al. developed an MRI/fluorescence bimodal platform for detecting GSH using MnO_2_ nanosheets. These nanosheets serve as a DNA nanocarrier, fluorescence quencher, and GSH-activated MRI CAs [27]. Upon endocytosis, the nanosheets react with GSH, releasing Mn^2+^ ions for MRI and primers for RCA, enhancing fluorescence, offering high selectivity for analyzing cellular GSH levels.

### 2.3. Fe-Based CAs

Like Mn ions, Fe ions possess multiple unpaired electrons (five for Fe^3+^ and four for Fe^2+^), making them suitable for T_1_-MRI. Fe-based CAs exhibit superior biocompatibility compared to Gd-based or Mn-based alternatives, as iron is naturally found in human blood, mainly within ferritin. Various low molecular weight Fe-chelates have been synthesized for T_1_-MRI applications [28]. Xie et al. created [Fe(tCDTA)]^−^ and its derivatives with superior T_1_-MRI performance using a straightforward two-step synthesis. The results of 3T MRI tests suggested the r_1_ value of these Fe-chelates ranged from 2.06 to 3.53 mM^−1^s^−1^, which is comparable to commercial Gd-chelates [29].

In addition to enhancing stability and half-life, there is growing interest in developing IONPs-based T_1_ CAs. This approach addresses the limitation of the narrow MRI time frame found in chelates. Five types of IONPs have been approved and used clinically [30]: Ferumoxides (Feridex^®^, Bayer HealthCare Pharmaceuticals), Ferucarbotran (Resovist^®^, Bayer Schering Pharma AG), Ferumoxtran-10 (AMI-227 or Code-7227, Combidex^®^, AMAG Pharma; Sinerem^®^, Guerbet), NC100150 (Clariscan^®^, Nycomed), and VSOP C184 (Ferropharm). Clariscan^®^ and VSOP C184 were designed for MR angiography and blood pool imaging; Combidex^®^ and Sinerem^®^ for lymph node imaging; and Feridex^®^ and Resovist^®^ for liver imaging. These agents received regulatory approval in various countries. For now, although most of these products have been removed from the market for economic reasons (only Resovist^®^ is currently available in limited countries), IONPs still show promising applications for MRI. The details of the IONPs used in T_1_-MRI are reviewed in the subsequent sections of this paper.

### 2.4. Nanodiamond-Based CAs

Nanodiamonds (NDs) have garnered interest as a biocompatible alternative to other carbon nanomaterials, thanks to their lower tendency to induce cellular oxidative stress [31]. Initial research centered on their use as nontoxic drug delivery vehicles and stable fluorescent markers for cell tracking [32,33,34]. Recently, there has been a surge in exploring nanodiamonds for enhancing MRI contrast.

Research by Manus and colleagues demonstrated that conjugating ND with Gd^3+^ can significantly boost T_1_-MRI contrast [35]. The per-Gd r_1_ value reached 58.82 ± 1.18 mM^−1^s^−1^ at 1.5 T, a tenfold improvement over the monomer Gd complex (r_1_ = 5.42 ± 0.20 mM^−1^s^−1^). However, high intracellular accumulation of Gd^3+^-ND conjugates led to a negative contrast by shortening T_2_, obscuring the positive contrast from T_1_ shortening and making labeled cells barely discernible on T_1_-weighted images. Furthermore, the long-term safety of Gd^3+^-ND conjugates is still a concern due to the potential release of Gd^3+^. Lazovic and team created unique detonation ND with paramagnetic centers, allowing effective detection in T_1_- MRI images at biologically relevant concentrations without requiring Gd conjugation [36]. This advancement streamlines both detection and production processes. Their r_1_ value at 7T was 11.26 mM^−1^s^−1^, nearly triple that of commercial gadobutrol, showcasing promising potential for Gd-free MRI applications.

## 3. Preparation Methods of IONPs for T_1_-MRI

The T_1_-MRI effectiveness of IONPs is greatly influenced by their composition, structure, size, and morphology. Thus, selecting a suitable synthesis method is crucial for the controlled fabrication of high-performance IONPs. Various synthetic strategies have been used to control nanoparticle growth kinetics by modifying reaction temperatures or using different ligands, such as chemical co-precipitation, solvothermal methods, thermal decomposition, and microemulsion methods.

### 3.1. Co-Precipitation Methods

**Figure 1:**
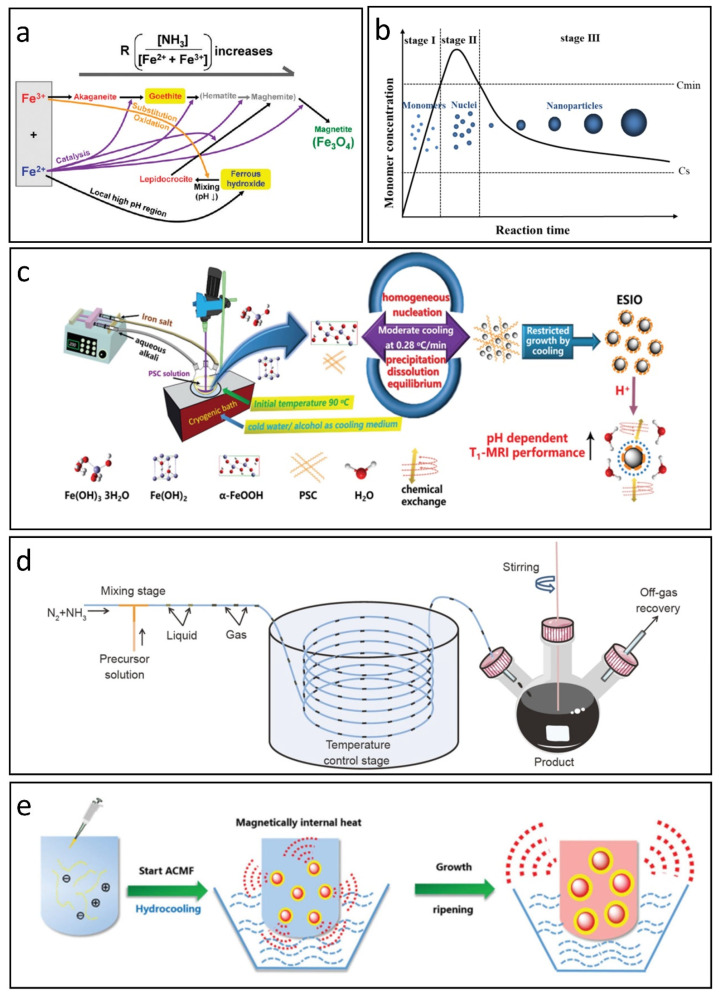
Co-precipitation methods for synthesizing IONPs. (**a**) The reaction pathway for the preparation of IONPs via co-precipitation. Reproduced with permission [38]. Copyright 2012, American Chemical Society. (**b**) LaMer’s mechanism illustrating the nucleation and growth of nanoparticles. Reproduced with permission [39]. Copyright 2015, American Chemical Society. (**c**) Moderate cooling co-precipitation technique. Reproduced with permission [40]. Copyright 2020, The Royal Society of Chemistry. (**d**) Co-precipitation method utilizing a gas/liquid mixed-phase fluidic reactor. Reproduced with permission [41]. Copyright 2022, Springer Nature. (**e**) Magnetically induced internal heating co-precipitation approach. Reproduced with permission [42]. Copyright 2018, The Royal Society of Chemistry.

The aqueous co-precipitation technique is widely recognized as the primary method for producing IONPs. In this process, Fe^2+^ and Fe^3+^ salts co-precipitate in a basic solution with a molar ratio of 1:2, either at room temperature or with heating [37]. This approach is both convenient and cost-effective, facilitating rapid large-scale production of nanoparticles that remain dispersed in an aqueous medium, making them suitable for biomedical applications without complex ligand exchanges [19]. Generally, IONPs are synthesized within a pH range of 8 to 14 through the hydrolysis of Fe^2+^ and Fe^3+^. The intricate nature of the hydrolysis reactions contributes to the emergence of various intermediate phases, requiring careful management of factors like base concentration, temperature, the ratio of Fe^2+^/Fe^3+^, ionic strength, and surfactants to optimize the nanoparticles’ size, shape, composition, and magnetic properties. Ahn et al. found that magnetite nanoparticles are formed during co-precipitation through the phase transformation of iron oxyhydroxides, not by direct reactions of Fe^2+^ and Fe^3+^. Akaganeite transforms into goethite, which topotactically converts to magnetite as pH increases. Meanwhile, ferrous hydroxide transforms into lepidocrocite, which also converts to magnetite upon rapid mixing with the base solution, mediated by arrow-shaped nanoparticles. These transformations correlate with specific crystallographic relationships among iron oxides and occur via an oriented aggregation mechanism, see Figure 1a [38].

Although controlling the above reaction conditions is commonly employed to produce IONPs with various sizes and morphologies, the co-precipitation reaction is thermodynamically driven; this reliance complicates the regulation of size distribution and crystallinity, which are crucial for their biomedical efficacy. Recent research has increasingly focused on optimizing reaction kinetics, especially managing the nucleation and growth phases of IONPs. Vreeland et al. introduce a general method for precise size control in IONP synthesis by maintaining steady growth conditions through continuous, controlled precursor addition, resulting in a uniform growth rate. This approach, termed the “Extended LaMer Mechanism”, enables reproducibility in particle size across batches and allows for size prediction by monitoring early growth stages (Figure 1b) [39]. Chen et al. proposed a chemical co-precipitation technique that utilizes a gentle, controlled cooling process to promote uniform nucleation and slow growth, resulting in monodispersed IONPs averaging 3.43 nm in size (Figure 1c) [40]. These IONPs exhibit approximately three times higher T1-MRI signal intensity compared to commercial Ferumoxytol, similar to that of Gd-based CAs in vitro.

In addition, ensuring uniform mixing and heating of precursors is essential for achieving monodispersed IONPs. Mao et al. proposed a gas/liquid mixed-phase fluidic reactor that employs NH_3_ to establish alkaline conditions favorable for co-precipitation, see Figure 1d [41]. This innovative approach facilitates rapid and even mixing of alkali with iron ions, producing monodispersed IONPs with average sizes from 1.78 nm to 4.04 nm. The synthesized IONPs demonstrate superior T_1_ MRI contrast effects in water, cellular environments, and blood vessels. Furthermore, Chen et al. enhanced the conventional co-precipitation technique by integrating hydro-cooling and magnetically induced internal heating within an alternating magnetic field, as shown in Figure 1e [42]. This form of self-heating may transition the colloidal growth from a thermodynamic to a kinetic regime, allowing for more complete reactions and improving the crystallization and uniformity of the resulting IONPs.

### 3.2. Solvothermal Methods

When traditional aqueous methods yield nanoparticles with inadequate crystallization, the solvothermal technique offers an alternative by substituting water with organic solvents. This results in monodisperse IONPs with enhanced crystallinity and defined shapes. Although effective for controlling size and morphology, this method often requires much longer processing times, ranging from several hours to days [43]. Solvents such as polyols and polyethylene glycol are advantageous due to their high dielectric constants, which aid in dissolving inorganic compounds and provide a broad operational temperature range (from 25 °C to their boiling points) [44]. These solvents sometimes act as both reducing and stabilizing agents, facilitating controlled particle growth and preventing aggregation. Several factors—including solvent type, salt ratios, concentrations, and other physical conditions—significantly affect the development, shape, size, and yield of nanoparticles.

Zheng et al. introduced a novel method for creating monodispersed Fe_3_O_4_ nanoparticles by integrating oxidation precipitation with solvothermal synthesis [45]. Their process produced nanoparticles with average sizes between 5 and 12 nm. The study explored how different surfactants, alcohols, and the amounts of alkaline and surfactants influenced the size, morphology, and dispersion of the nanoparticles, leading to optimal reaction conditions. Moreover, adjusting the amount of alkaline resulted in various phases of the product, including Fe_3_O_4_, α-Fe_2_O_3_, γ-Fe_2_O_3_, and α-FeOOH. Luo et al. created stable 2.7 nm Fe_3_O_4_ nanoparticles via a solvothermal method, functionalized with PEG-RGD [46]. These nanoparticles show excellent water dispersibility, stability, cytocompatibility, and hemocompatibility, as well as specific targeting of glioma cells overexpressing αvβ3 integrin in vitro. Their superior T_1_-MRI performance enables effective targeted imaging of glioma cells in vitro and in vivo.

### 3.3. Thermal Decomposition

Thermal decomposition is a proven method for synthesizing high-quality and monodispersed IONPs [47,48,49]. Typically, iron precursors such as Fe(CO_)5_ or Fe(acac)_3_ are heated to high temperatures in the presence of growth inhibitors (like oleyl alcohol or hexadecanediol), reductants, surfactants (such as oleic acid or octadecylene), and organic solvents. This technique shows significant advantages over aqueous methods, particularly in producing ultra-small IONPs (less than 5 nm) [12]. The reaction temperature for these nanoparticles is usually maintained between 200 and 250 °C, with the growth inhibitor added to slow down further growth. Rapidly quenching the reaction after nucleation prevents excessive particle growth, which is essential for separating nucleation from growth. This separation helps minimize complex hydrolysis reactions, resulting in high yields and reduced aggregation. By adjusting solvent types, surfactant ratios, and heating parameters (including temperature and duration), it is possible to achieve monodisperse IONPs with controlled shapes and sizes.

**Figure 2:**
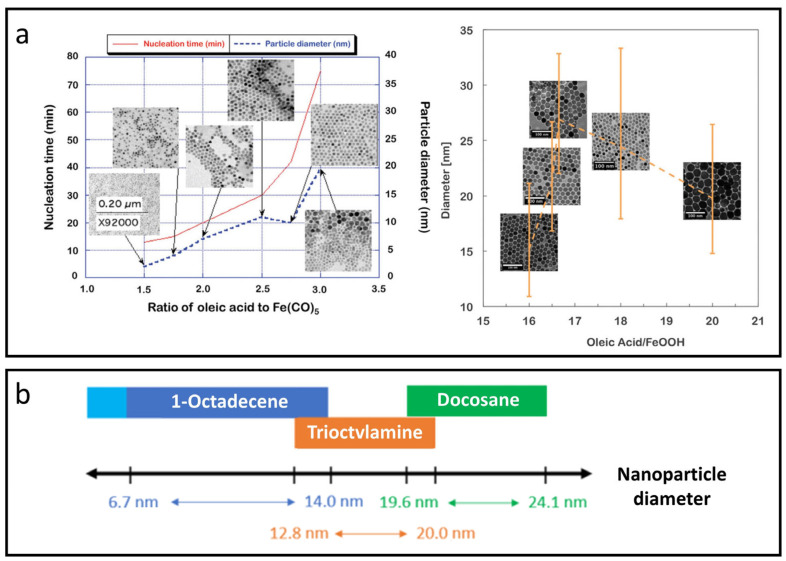
Thermal decomposition methods for synthesizing IONPs. (**a**) Excess surfactant (oleic acid) modifies the reaction kinetics, resulting in larger particles with uniform sizes. Reproduced with permission [50]. Copyright 2015, The Royal Society of Chemistry. (**b**) Chart of suitable solvents for achieving a wide range of IONP diameters with narrow size distribution, high crystallinity, and reproducible size control within ±0.5 nm. Reproduced with permission [51]. Copyright 2022, American Chemical Society.

Hufschmid et al. explored critical factors for synthesizing monodisperse IONPs through thermal decomposition, evaluating three different iron precursors: iron oleate, iron pentacarbonyl, and iron oxyhydroxide, under various conditions [50]. They produced IONPs with sizes ranging from approximately 2 to 30 nm by fine-tuning parameters such as precursor concentration, surfactant addition, temperature, ramp rate, and reaction time. Notably, using a large excess of surfactant (up to a 2.5:1 molar ratio) altered the reaction kinetics, leading to larger particles with uniform sizes (Figure 2a), although this often resulted in a trade-off between particle size and distribution uniformity. The iron oxide phase, along with nanoparticle size and shape, plays a vital role in determining magnetic properties like differential susceptibility and anisotropy.

Fokina et al. found that specific solvents, including 1-octadecene, trioctylamine, and docosane, facilitated temperature ranges from starting points of 100 °C to maximum temperatures of up to 370 °C, enabling the synthesis of monodisperse nanoparticles ranging from 6 to 24 nm in diameter on a large scale [51]. Interestingly, each solvent corresponds to a specific temperature range, allowing reproducible control of nanoparticle size to within ±0.5 nm through variations in temperature, heating rates, ligand types, or precursor concentrations, as shown in Figure 2b. Kim et al. synthesized uniform ultra-small IONPs, less than 4 nm in size, using thermal decomposition with oleyl alcohol [52]. In vivo T1-weighted MRI demonstrated that these ultra-small IONPs have a longer circulation time than clinical gadolinium-based agents, enabling detailed imaging. Blood pool MR imaging with USIONPs clearly revealed vessels as small as 0.2 mm.

The advantages of thermal decomposition methods allow for IONPs that exhibit enhanced relaxivity and saturation magnetization, making them suitable for various biomedical applications. However, it is crucial to recognize that IONPs created through this method are typically insoluble in water, requiring additional modifications to improve their compatibility with biological solutions [53].

### 3.4. Microemulsion Methods

The microemulsion technique employs a stable oil–-water mixture combined with surfactants to create a controlled environment for synthesizing nanoparticles [54,55,56]. In water-in-oil microemulsions, nano-sized water droplets are dispersed within the oil phase and stabilized by surfactants, effectively preventing particle growth and aggregation. Researchers are actively investigating different surfactant combinations to enhance the synthesis process, yielding nanoparticles with core diameters ranging from 8 to 16 nm and shell sizes between 2 and 3 nm. For instance, Okoli et al. synthesized IONPs in two distinct microemulsion systems using a cationic surfactant (CTAB) and a nonionic surfactant (synperonic 10/6) [57]. The IONPs produced with CTAB exhibited a larger size (9.2 nm) compared to those made with synperonic 10/6 (2.2 nm), which significantly affected their magnetization properties. Additionally, factors such as the concentration of Fe^2+^/Fe^3+^ ions, temperature, and pH levels were found to play crucial roles in controlling the size of the IONPs [58]. Liu et al. developed a pH-sensitive T_2_-T_1_ switchable MRI nanoprobe through a combination of microemulsion and biomineralization, resulting in CaCO_3_-coated PEG-IONPs [59]. This nanoprobe offers uniform and controllable size with a straightforward preparation method. Upon exposure to acidic environments, such as those in tumor tissues, the CaCO_3_ coating dissolves, freeing PEG-USPIONs for T_1_-weighted tumor imaging. The nanoprobe’s effectiveness was validated in a mouse model bearing a 4T1 xenograft.

In this method, the microemulsion bubbles act as controlled “microreactors”, resulting in typically spherical structures with uniform sizes, and the production of IONPs that often exhibit superparamagnetic properties and high magnetization levels [43]. However, a notable drawback of this method is that residual surfactants can adversely affect the material properties [60,61], and scaling up the production remains challenging.

## 4. Theoretical Fundamentals of IONPs for T_1_-MRI CAs

The MRI process begins with a static magnetic field that aligns or counter-aligns the spins of hydrogen protons. This alignment is followed by the application of a radiofrequency pulse, which excites these spins [62]. The subsequent recovery processes include T_1_ (longitudinal) relaxation, where spins release energy to align or counter-align with the magnetic field, and T_2_ (transverse) relaxation, during which spins lose phase coherence in the transverse plane [63]. To enhance the contrast in MRI images, the interaction between CAs and surrounding water protons is vital, as it shortens the T_1_ and T_2_ relaxation times of nearby water molecules. T_1_ CAs increase signal intensity, resulting in brighter T_1_-weighted images, while T_2_ CAs decrease signal intensity, yielding darker T_2_-weighted images. The effectiveness of these agents is evaluated through relaxivity parameters: r_1_ is longitudinal relaxivity and r_2_ is the transverse relaxivity, indicating their potential for bright and dark signal enhancement, respectively [64]. The ratio r_2_/r_1_ helps differentiate between T_1_ and T_2_ CAs. Typically, T_1_ agents have a lower ratio, ranging from 1 to 2, while T_2_ agents exhibit a higher ratio that exceeds 8; ratios between 2 and 8 are generally classified as dual-mode CAs. For IONP-based CAs, the electronic structure of iron ions is essential for their MRI enhancement capabilities. Fe^3+^ and Fe^2+^ ions in a high spin state exhibit spin values of S = 5/2 and S = 2; their spin-only magnetic moments are 5.9 μ_B_ and 4.9 μ_B_, respectively, both of which indicate an optimal electronic structure for T1 CAs [3,65].

The relaxation of water protons near an ion center is generally described by the inner sphere, secondary sphere, and outer sphere mechanisms [66,67]. Inner sphere relaxation arises from water molecules that are directly coordinated to the paramagnetic center [68,69], whereas outer sphere relaxation is influenced by bulk water in the surrounding environment [70]. In certain situations, water molecules bonded through hydrogen interactions with chelators are considered part of the secondary sphere [71]. On the base of the above mechanisms, Solomon-Bloembergen-Morgan (SBM) theory and quantum mechanical outer sphere theory were proposed for T_1_ and T_2_ CAs, respectively.

### 4.1. SBM Theory

**Figure 3:**
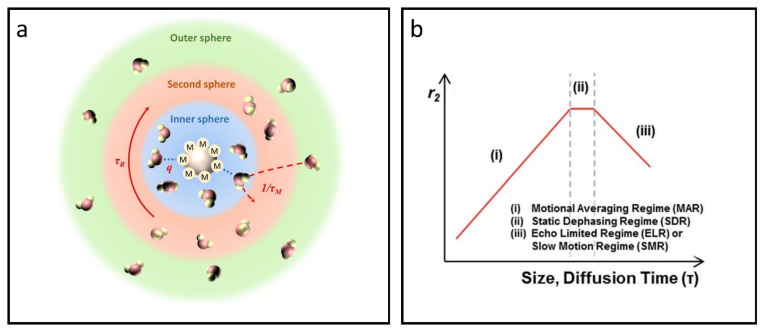
Overview of the SBM theory and quantum mechanical outer sphere theory. (**a**) The impact of the inner sphere, second sphere, and outer sphere on the T_1_ relaxation process according to SBM theory. (**b**) Size- and diffusion time-dependent T_2_ relaxivity as predicted by outer sphere relaxation theory, featuring three distinct regimes: (i) MAR, (ii) SDR, and (iii) ELR. Reproduced with permission [80]. Copyright 2015, The Royal Society of Chemistry.

According to SBM theory, T_1_ CAs cause energy loss through dipole–-dipole interactions between water protons and magnetic ions, and their interaction regions can be divided as inner-sphere, secondary intermediate sphere, and outer sphere [72,73], as shown in Figure 3a. Among them, the T_1_ relaxation enhancement is mainly originated from the inner sphere. The r_1_ value in the inner sphere can be characterized by several parameters, including the mole fraction of coordinating water (*P_m_*), applicable dipole–-dipole relaxation (*T_1m_*), coordination number of water (*q*), correlation time (*τ_c_*), proton residence lifetime (*τ_m_*), molecular tumbling time (*τ_R_*), electronic relaxation time (*T_1e_*), proton Larmor frequency (*ω_H_*), and the distance between magnetic ions and protons (*r_H_*); see the following equations [74,75,76].

(1) $$r_{1}=\frac{qP_{m}}{T_{1m}+\tau_{m}}$$

(2) $$\frac{1}{T_{1m}}=\frac{2}{15}(\frac{\mu_{0}}{4\pi })[\frac{\gamma_{H}^{2}g_{e}^{2}\mu_{B}^{2}S(S+1)}{r_{H}^{6}}][\frac{3\tau_{c}}{1+\omega_{H}^{2}\tau_{c}^{2}}]$$

(3) $$\frac{1}{\tau_{c}}=\frac{1}{\tau_{m}}+\frac{1}{\tau_{R}}+\frac{1}{T_{1e}}$$

The equations above also include several important constants. *μ_B_* represents the Bohr magneton constant, *γ_H_* denotes the gyromagnetic ratio of the proton, *g_e_* is the electron g-factor, and *S* signifies the spin quantum number for the respective ion species.

The performance of CAs is determined by time parameters that describe the dynamics of water in different environments, the rotational movement of the CAs, and specific relaxation mechanisms [77]. These parameters are influenced by the strength of the external magnetic field, the molecular configuration of the CAs, and their physical and chemical characteristics at the interface with water. As a result, they have a complex impact on the relaxivity of the CAs. In Equation (3), the term 1/τ_c_ is primarily influenced by the largest of the three terms, indicating that τ_c_ is most significantly impacted by the shortest correlation time. When operating at field strengths of 1.5 T or above, electronic relaxation slows down considerably, as T_1e_ increases with the square of the magnetic field strength [78,79]. At this stage, relaxivity becomes more dependent on either the rotational motion (1/τ_R_) or the water exchange rate (1/τ_m_).

For nanoparticle-based CAs, the value of τ_R_ tends to be much longer than that of τ_m_, meaning that τ_m_ becomes the critical factor in enhancing T_1_ contrast effectiveness. The range of τ_m_ can vary from 0.1 ns to several microseconds, depending on the local coordination conditions. When considering inner -sphere relaxation, τ_m_ is typically much shorter than T_1m_, which means water molecules can often be released before achieving full relaxation [77]. For nanoparticle-based CAs, it is possible to adjust the chemical environment to prolong τ_m_ for improved r_1_. However, careful consideration is needed, as excessively prolonging water exchange may adversely affect the release rate of relaxed water molecules, ultimately compromising the relaxation effect in bulk water.

### 4.2. Quantum Mechanical Outer Sphere Theory

The outer sphere theory was initially utilized to study relaxation behaviors in weakly magnetized particles, highlighting how water diffusion affects relaxation processes [81]. T_2_ relaxation arises mainly from spin-spin interactions that occur during water diffusion, affirming the applicability of quantum mechanical outer sphere theory to magnetic nanoparticles. Recent advancements in nanotechnology indicate that high magnetic moments in magnetic or superparamagnetic IONPs can generate localized inhomogeneous magnetic fields when exposed to an external magnetic field. This effect disrupts phase coherence among adjacent water molecules and enhances T_2_ relaxation for protons in the vicinity [82,83].

Proton dephasing can be categorized into three stages based on the water diffusion time *τ_D_*: the motional average regime (MAR), the static dephasing regime (SDR), and the echo-limiting regime (ELR) [80,84,85], see Figure 3b. The value of *τ_D_* can be determined using the formula *τ_D_ = d^2^*/4*D*, where *d* represents the effective radius of the particle and *D* is the diffusivity of water molecules. When the size of IONPs is sufficiently small, *τ_D_* becomes less than 1/(*γ_H_B_eq_*)—where *B_eq_* represents the equatorial magnetic field—resulting in the fulfillment of the MAR condition. During this phase, protons move rapidly around the IONPs, encountering rapid fluctuations in the magnetic field, leading to effective time-averaging within the MAR stage. The r_2_ of IONPs can be expressed using the following equation [81].

(4) $$r_{2}=\frac{(256\pi^{2}{\gamma_{H}}^{2}/405)V^{*}M_{S}r^{2}}{D(1+L/r)}$$

This particular equation incorporates the effective radius *r*, saturation magnetization *M_S_*, effective volume fraction V*, and the thickness of the impermeable coating on the magnetic core *L*. It is important to highlight that T_2_ contrast in IONPs shows a positive association with both M_S_ and *r*. Larger IONPs generally demonstrate higher M_S_ as a result of the spin canting effect; thus, enlarging the nanoparticles emerges as an effective approach to bolster T_2_ relaxation. When IONPs exceed a certain size, proton relaxation around these larger particles is dominated by the SDR [86]. In this regime, protons experience complete dephasing before they diffuse a critical distance to encounter a significantly different magnetic field, assuming the particles are sufficiently large or the magnetic field strength is adequate. The r_2_ value for IONPs attains its maximum, as detailed below.

(5) $$r_{2}=\frac{8\sqrt{3}\pi^{2}}{81}\frac{A^{3}N_{0}}{10^{6}Z}\gamma_{H}M_{S}$$

In this equation, *A* denotes the lattice parameter, *N_0_* refers to Avogadro’s constant, and *Z* indicates the number of formula units within a unit cell. This relationship shows that the r_2_ value of IONPs in SDR is closely linked to its M_S_.

As the size increases, the T_2_ relaxivity of IONPs approaches ELR, at which point the relaxation becomes unaffected by particle size and echo time in the measurements [87]. This unwanted characteristic results in a decrease in T_2_ contrast as the size grows. Such behavior is primarily documented through computer simulations and has been sporadically noted in magnetic clusters under specific extreme conditions [88,89].

## 5. Factors Influencing T_1_-MRI Enhancement of IONPs

### 5.1. Size

**Figure 4:**
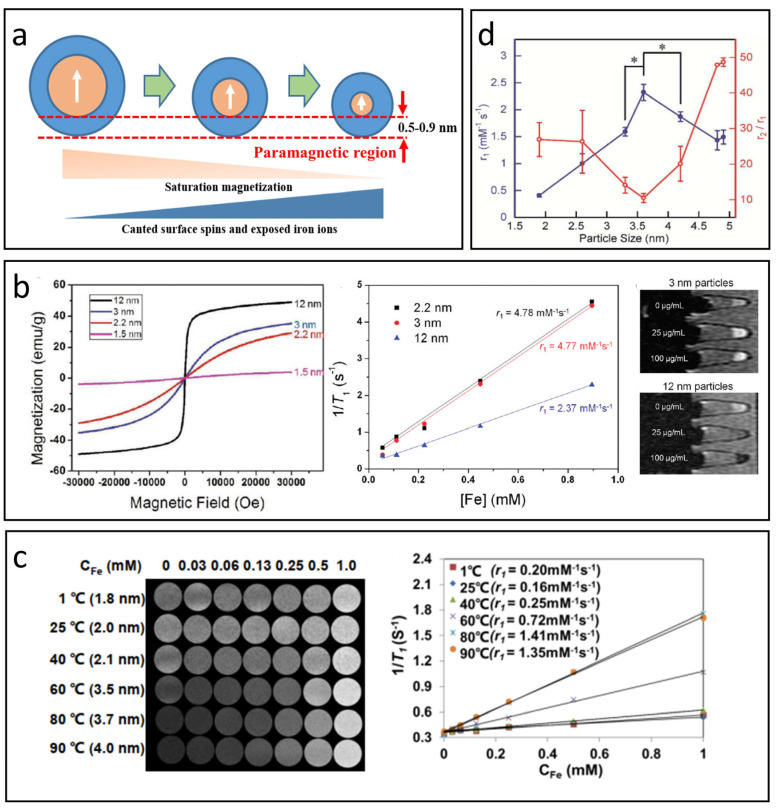
The effect of nanoparticle size on T_1_-MRI performance. (**a**) The impact of IONP size on magnetization, canted surface spins, and exposed iron ions. (**b**) Variation in magnetic properties and T_1_ relaxation properties with respect to IONP size. Reproduced with permission [52]. Copyright 2011, American Chemical Society. (**c**) Correlation between r_1_ value and nanoparticle size as reported in Mao’s study. Reproduced with permission [41]. Copyright 2022, Springer Nature. (**d**) The r_1_ value and r_2_/r_1_ ratio across different nanoparticle sizes (mean ± SD, n = 3, * *p* < 0.02) based on Shen’s research. Reproduced with permission [95]. Copyright 2017, American Chemical Society.

According to the SBM theory (Equation (1)), the number of bound water molecules *q* is directly related to the r_1_ value of T_1_ CAs [18]. For IONPs, their ability to shorten T_1_ relaxation stems from the iron ions present on their surface. Since the *q* value for these iron ions is constant, a practical way to enhance *q* in IONPs is to increase the number of iron ions on the surface, effectively improving the surface-to-volume ratio (S/V) [90,91,92], see Figure 4a. Smaller IONPs usually exhibit greater T_1_ contrast enhancement because their S/V increases as their size decreases.

The ratio of r_2_/r_1_ is a crucial indicator for assessing whether a CA is appropriate for positive (T_1_) or negative (T_2_) imaging, highlighting the importance of examining how r_2_ varies with nanoparticle size [12]. According to quantum mechanical outer sphere theory, for smaller IONPs achieving the MAR, water diffusion around IONPs occurs much faster than the resonance frequency shifts, resulting in lower r_2_ values as IONP size decreases. Additionally, a reduction in IONP size can weaken the magnetic moment, further contributing to lower r_2_ values. This phenomenon can be understood through the spin -canting effect [93,94]: disruptions in the crystal structure lead to disordered spins on the nanoparticle surface. These surface spins do not align perfectly with the bulk spins, resulting in a paramagnetic region at the surface. As IONPs become smaller, a larger fraction of their volume consists of this paramagnetic region. For example, when the iron oxide core measures 5 nm, about half of its volume is this paramagnetic region, increasing to 78% for 2.5 nm [5].

As previously mentioned, decreasing the size of IONPs can enhance T_1_ relaxation while diminishing T_2_ relaxation, effectively improving the T_1_ contrast capabilities of these nanoparticles. Kim et al. successfully created uniform IONPs with diameters under 12 nm through a controlled thermal decomposition process of iron–-oleate complexes in oleyl alcohol [52]. Figure 4b illustrates that the magnetization of the synthesized IONPs is positively correlated with their size, attributed to the spin canting effect. Additionally, smaller IONPs generally have a higher S/V, leading to an increased number of surface Fe^3+^ ions and consequently a greater r_1_ value. The r_1_ value rose from 2.37 mM^−1^ S^−1^ to 4.78 mM^−1^ S^−1^ as the size decreased from 12 nm to 2.2 nm, indicating that ultra-small IONPs can serve as effective T_1_ CAs.

It is important to recognize that T_1_ contrast in IONPs does not consistently improve as nanoparticle size decreases. Research has shown that as IONPs become larger, τ_R_ significantly increases, becoming the primary factor that enhances the r_1_ value [96]. Mao et al. developed small IONPs with diameters less than 4 nm using a gas/liquid mixed-phase fluidic reactor combined with co-precipitation, carefully adjusting the temperature to control the size (see Figure 4c) [41]. Tests conducted with a 3T MRI scanner revealed that the r_1_value of the IONPs rises initially and then declines as their size decreases, reaching a maximum at around 3.7 nm, where it presents a high r_1_ value of 4.11 mM^−1^ s^−1^ and a low r_2_/r_1_ratio of 7.90, indicating excellent T_1_-MRI contrast performance in water, cellular environments, and blood vessels. A similar trend was observed in Shen’s study (Figure 4d) [95], which reported a peak at 3.6 nm, yielding an r_1_ value of 2.32 mM^−1^ s^−1^ and a low r_2_/r_1_ ratio of 10.5 under 7T scanner.

### 5.2. Morphology

The morphology of IONPs is just as crucial as their size in influencing their properties. Although spherical IONPs are the most extensively studied CAs, variations in morphology can affect both T_1_ and T_2_ relaxivities [17]. An optimal morphology for T_1_-MRI should strike a balance to achieve a favorable r_2_/r_1_ ratio.

**Figure 5:**
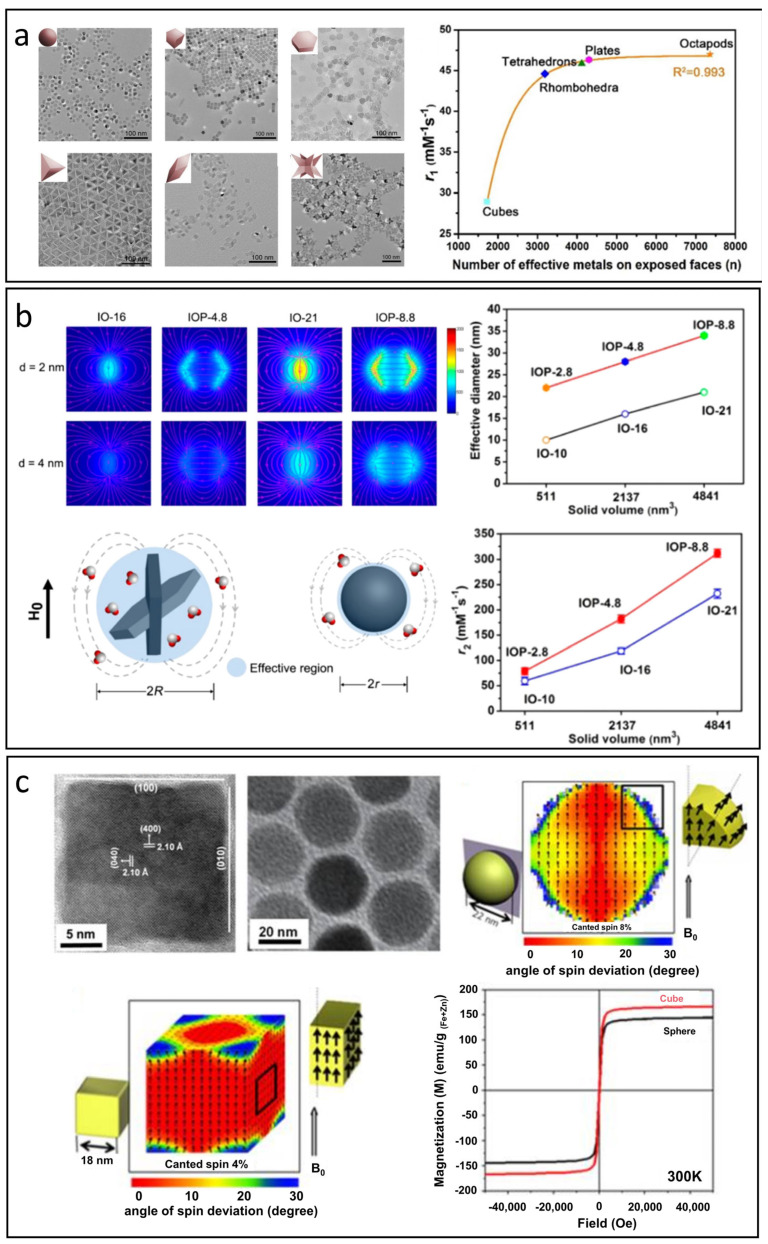
The effect of morphology on T_1_ and T_2_ relaxation processes. (**a**) The correlation between S/V, the number of effective metal ions on exposed surfaces, and the r_1_ value. Reproduced with permission [97]. Copyright 2018, American Chemical Society. (**b**) The influence of effective diameter on stray fields and the r_2_ value in nanoplates compared to spheres. Reproduced with permission [98]. Copyright 2014, American Chemical Society. (**c**) Magnetic spin configurations of cubes and spheres and their resulting magnetic properties. Reproduced with permission [99]. Copyright 2012, American Chemical Society.

The r_1_ value of nanoparticles is primarily influenced by the S/V, with higher values linked to a greater number of effective metal ions on exposed surfaces. This count depends on both surface area and the specific crystal facets revealed [91,97]. Yang et al. synthesized manganese-doped IONPs with six morphologies: spheres, cubes, plates, tetrahedrons, rhombohedra, and octapods (Figure 5a) [97]. The orientation of these facets impacts T_1_ relaxivity; for instance, the (100) facet has only two effective Fe^3+^ ions, while the (110) facet contains more. Thus, cubes with (100) facets exhibit lower r_1_values, whereas rhombohedra show intermediate ones. Higher r_1_values in plates and tetrahedrons are due to their (111) and (110) surfaces providing more ions for exchanges with water protons. Octapods, however, display lower r_1_values, likely due to their less defined (311) surfaces. Overall, r_1_ values correlate positively with the total number of effective metal ions across exposed facets for each morphology.

The effect of morphology on the r_2_ value is primarily related to the effective radius when the M_s_ values are comparable. Different nanoparticle shapes possess unique effective radii, which significantly influence the intensity of stray fields and local field inhomogeneity caused by IONPs, which ultimately affects water diffusion processes and T_2_ relaxation behavior. Zhou et al. highlighted that nanoplates outperform the nanospherical morphology in T_2_ relaxation properties [98]. Due to their rapid random flipping in the medium, nanoplates with anisotropic shapes can be effectively treated as simulated spheres based on their edge lengths. Consequently, the effective radius of a nanoplate is considerably larger than that of a sphere with an equivalent solid volume, leading to much greater local field inhomogeneity. As illustrated in Figure 5b, the r_2_ values for nanospheres are significantly lower than those for the corresponding nanoplates, likely due to the larger effective diameters of the nanoplates.

Furthermore, variations in morphology also impact the orientation of spins on the surface of IONPs, which in turn affects both magnetization and the r_2_ value. Noh et al. discovered that the disordered spins are evenly distributed across the surfaces of spherical IONPs with a specific thickness. In contrast, for cubic IONPs, the disordered spins tend to concentrate at the corners, see Figure 5c [99]. The proportion of disordered spins in cubes is approximately 4%, notably lower than the 8% observed in spheres, leading to a significantly higher magnetization in cubic IONPs.

### 5.3. Surface Modification

To prevent aggregation and improve water dispersion and biocompatibility, the surface of IONPs must be coated with hydrophilic materials such as small molecules, polymers, or proteins [100]. This modification is essential for enhancing MRI contrast because it greatly affects how IONPs interact with water molecules.

**Figure 6:**
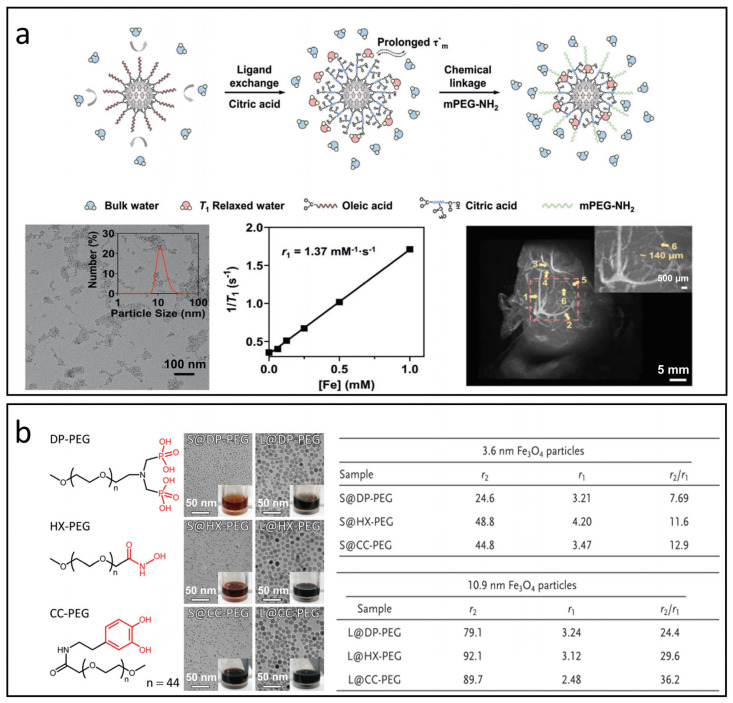
The impact of surface modifications on T_1_ and T_2_ relaxation processes. (**a**) IONPs modified with citric acid and mPEG-NH_2_ to enhance τ_m_ for 7 T MRI applications. Reproduced with permission [101]. Copyright 2021, John Wiley & Sons, Ltd. (**b**) Relaxation properties of IONPs treated with PEG containing various anchoring groups: diphosphate (DP-PEG), hydroxamate (HX-PEG), and catechol (CC-PEG). Reproduced with permission [103]. Copyright 2014, John Wiley & Sons, Ltd.

For effective T_1_ relaxation, water molecules must bind directly to the iron centers; therefore, the T_1_ signal is highly sensitive to the type and concentration of the surface capping layer. These coatings not only influence the accessibility of iron ions but also affect water availability—both critical factors in determining the time parameters of the T_1_ relaxation process, including τ_R_ and τ_m_ [17,81]. Wang et al. modified ultra-small IONPs using citric acid and mPEG-NH_2_ to optimize τ_m_, see Figure 6a [101]. The resulting IONPs displayed an ultracompact hydrophilic surface, demonstrating superior T_1_ relaxation properties and enabling clear visualization of microvasculature as small as approximately 140 µm in diameter under 7T MRI. Leslie et al. enhanced the surface of IONPs with polyethylene glycol (PEG) in molecular weights of 550, 750, 1000, 2000, and 5000 [102]. Their findings revealed that the r_1_ value generally increased with higher molecular weights, primarily due to prolonged τ_R_. However, a slight decrease in r_1_ was observed when molecular weights exceeded 1000, attributed to thicker layers that hindered water access to iron ions.

Moreover, the anchoring groups of ligands that bond to Fe^2+^ or Fe^3+^ ions on IONPs are thought to influence their magnetization by modifying oxidation states and spin-canting [103,104,105]. This alteration may offer valuable insights for optimizing r_2_ values in IONPs intended as T_1_ CAs. Zeng et al. explored the impacts of anchoring groups of surface ligands on magnetic properties of Fe_3_O_4_ nanoparticles through the application of PEG2000 ligands with anchoring groups such as diphosphate (DP-PEG), hydroxamate (HX-PEG), and catechol (CC-PEG), as shown in Figure 6b [103]. Their findings indicate that the binding affinity of the surface ligand significantly correlates with the M_S_ of the Fe_3_O_4_ nanoparticles, ranking as follows: hydroxamate > catechol > diphosphate. The conjugated structure within the anchoring group can significantly enhance the T_2_ effect by increasing the inhomogeneity of the local magnetic field.

### 5.4. Valence State

The primary valence states in IONPs are divalent (Fe^2+^) and trivalent (Fe^3+^), with Fe^2+^ having four unpaired electrons and Fe^3+^ possessing five. Compared to Fe^2+^, Fe^3+^ has a higher spin value (S = 5/2) and spin-only magnetic moments of 5.9 μ_B_, which can result in larger r_1_ and r_2_ values. In addition, according to SBM theory, magnetic nanoparticles with extended *T_1e_* exhibit enhanced T_1_ relaxivity. Since Fe^2+^ has a much shorter *T_1e_* compared to Fe^3+^ (approximately 10^−9^ to 10^−11^ s for Fe^3+^ and 10^−12^ to 10^−13^ s for Fe^2+^) [106,107,108], this structural limitation reduces the effectiveness of relaxation enhancement in magnetite-based IONPs, constraining their use as T_1_ CAs. Consequently, substituting Fe^2+^ in magnetite with other magnetic ions that have longer *T_1e_* and more unpaired electrons could potentially mitigate this structural deficiency and boost the T_1_ relaxivity of magnetite [109].

**Figure 7:**
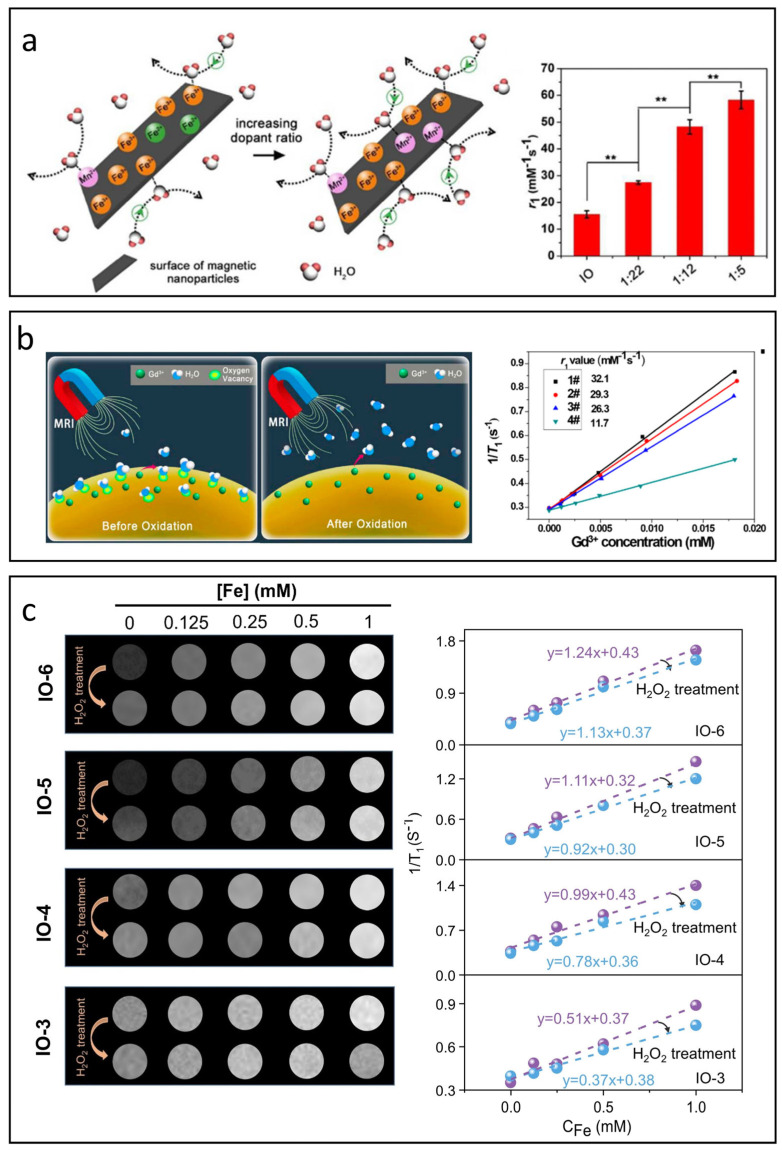
Influence of valence state and oxygen vacancies on T_1_ relaxation processes. (**a**) Effect of Mn^2+^ substitution on the T_1_ relaxivity of IONPs (** *p* < 0.01). Reproduced with permission [110]. Copyright 2018, The Royal Society of Chemistry. (**b**) Impact of oxygen vacancies on the r_1_value of Gd^3+^-based nanoparticles. Reproduced with permission [115]. Copyright 2017, American Chemical Society. (**c**) Effect of oxygen vacancies on the r_1_ value of IONPs. Reproduced with permission [116]. Copyright 2024, John Wiley & Sons, Ltd.

Zhao et al. replaced the undesirable Fe^2+^ ions with Mn^2+^ due to its longer *T_1e_* (10^−8^ s) and high spin value of S = 5/2 [110]. The findings revealed that these manganese ferrite nanoparticles exhibit significantly greater r_1_ values compared to their original IONPs, see Figure 7a. In addition to Mn^2+^, substituting Eu^2+^, which has seven unpaired electrons and a spin value of S = 7/2, also resulted in improved r_1_ values [111]. However, the highly reactive nature of Eu^2+^ can lead to rapid oxidation to Eu^3+^ (with spin value S = 3 and shorter T_1e_ compared to Eu^2+^), which not only reduces relaxivity but also increases toxicity [112]. This toxicity undermines the biocompatibility advantages associated with IONPs.

In IONPs, the ratios of Fe^2+^ and Fe^3+^ also influence the magnetic properties of IONPs, leading to variations in r_2_ values. Widely studied IONPs like magnetite and maghemite show either spinel or inverse spinel structures, featuring octahedral and tetrahedral sites [113,114]. Generally, Fe^3+^ has a strong inclination to occupy both types of sites, while Fe^2+^ mainly resides in octahedral positions. When subjected to an external magnetic field, the spins in the octahedral sites align parallel to the field, whereas those in the tetrahedral sites align antiparallel. These shifts in magnetic spin configurations theoretically affect both the magnetization and r_2_ values of IONPs.

### 5.5. Oxygen Vacancy

In the intricate process of nanoparticle synthesis, surface defects are unavoidable. Among these, oxygen vacancy defects are believed to significantly enhance the r_1_ value due to their inherent affinity for oxygen atoms in water molecules [115,117]. As shown in Figure 7b, Ni’s research was the first to incorporate oxygen vacancies into Gd^3+^-based nanoparticles, achieving an impressive r_1_ value of 32.1 mM^−1^s^−1^ on a clinical 3.0 T scanner [115]. In contrast to Gd^3+^-based nanoparticles, the role of oxygen vacancies in T_1_-MRI performance is more complex for IONPs. Zuo et al. studied the influence of oxygen vacancy on the T_1_ contrast performance of IONPs systematically [116], see Figure 7c. Beyond improving the affinity and adsorption of water molecules, oxygen vacancies also influence the valence states of iron ions. The formation of oxygen vacancies often leads to the generation of free electrons that can be drawn to nearby Fe^3+^ ions, reducing them to Fe^2+^. This transition decreases the number of unpaired electrons and shortens the electron relaxation time, resulting in lower r_1_ values. Additionally, changes in the valence state of iron ions can affect the magnetic coupling within sub-lattices, impacting both magnetization and r_2_ values.

## 6. Design of IONP-Based Responsive CAs

The modulation of the aforementioned factors has been widely utilized to influence the relaxation behavior of IONPs as T_1_ CAs, resulting in significant enhancements in T_1_-MRI contrast. However, traditional CAs produce MR signal enhancement regardless of their location, which results in limited target-to-background signal differences. Recently, responsive CAs have been introduced to respond to specific physiological changes or conditions, achieving better accuracy during diagnosis [118,119,120,121]. Common strategies for developing IONP-based responsive MRI CAs include “self-assembly/disassembly” and “distance adjustment”.

### 6.1. Self-Assembly/Disassembly

IONPs can exhibit adjustable MRI relaxivity through self-assembly or disassembly triggered by specific pathophysiological factors. This process modifies their magnetic properties and interactions with nearby water molecules, influencing their effectiveness in improving MRI contrast [122,123,124].

**Figure 8:**
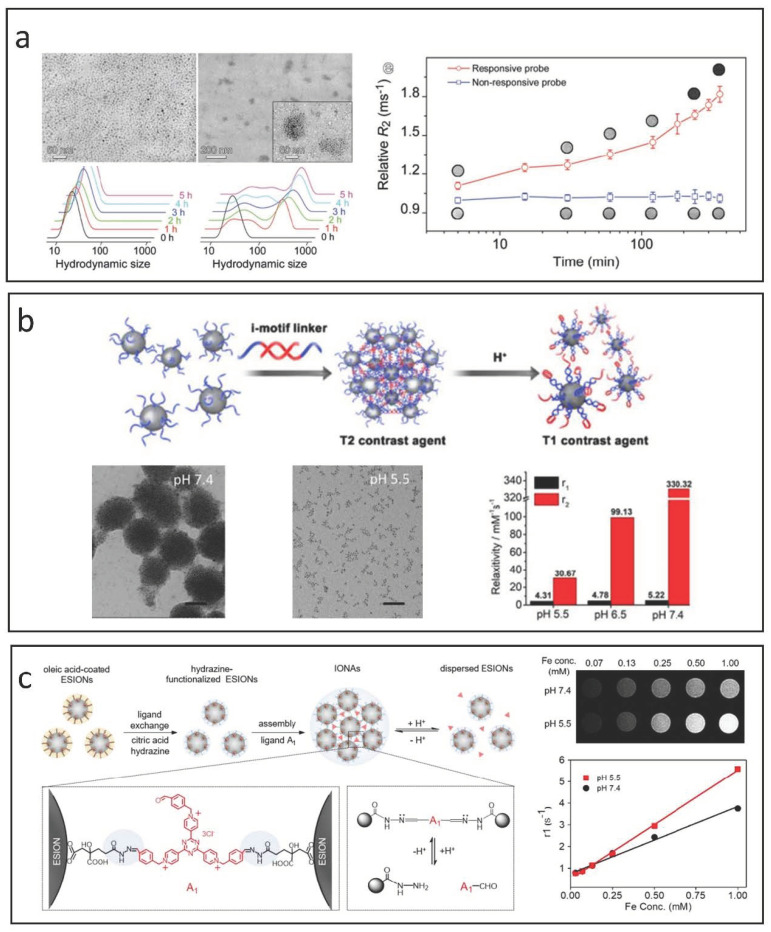
Strategies for designing IONP-based responsive CAs utilizing self-assembly and disassembly methods. (**a**) GSH-triggered T_1_/T_2_ switchable CAs using a self-assembly approach. Reproduced with permission [125]. Copyright 2017, John Wiley & Sons, Ltd. (**b**) pH-triggered T_2_/T_1_ switchable responsive CAs achieved through disassembly by incorporating i-motif DNAs. Reproduced with permission [126]. Copyright 2018, American Chemical Society. (**c**) pH-triggered T_2_/T_1_ switchable responsive CAs developed via disassembly through cross-linking with aldehyde derivative ligands. Reproduced with permission [127]. Copyright 2019, American Chemical Society.

The self-assembly of small IONPs influences r_1_ by altering the movement and distribution of water molecules within the system. While larger clusters may enhance T_1_ relaxation by increasing τ_R_ to better align with the Larmor frequency, the restricted mobility of water in these clusters can lead to a decreased r_1_ due to an increased τ_m_, moving it outside the optimal range [17]. Additionally, as cluster size increases and more IONPs occupy the less accessible inner areas, the contribution to r_1_ from these regions diminishes. Conversely, self-assembly is beneficial for improving T_2_ relaxation. The interactions with the magnetic fields generated by individual IONPs are significantly affected, which further enhances the inhomogeneity of the magnetic environment and accelerates dephasing, leading to higher r_2_ values. Thus, self-assembly strategies are often employed to create switchable responsive CAs for T_1_/T_2_ applications. For example, as illustrated in Figure 8a, Gao et al. developed GSH-triggered CAs by modifying IONPs with a PEG ligand that incorporates an RGD peptide along with a self-peptide sequence connected by a reducible disulfide bond [125]. When the self-peptide segment is cleaved by GSH in the tumor microenvironment, the in situ cross-linking of these responsive CAs can increase the tumor’s T_2_ contrast by more than threefold in vivo compared to non-cross-linkable IONP-based CAs.

The disassembly strategy generally transitions the contrast mode of IONPs from “T_2_” to “T_1_” in response to specific pathophysiological changes. To develop T_2_-mode MRI CAs, IONP-based nanoclusters or nanoaggregates are created using responsive ligands. In the bloodstream and healthy tissues, these agents remain as nanoclusters, but they break down into individual nanoparticles in the tumor microenvironment due to the acidic pH and elevated GSH levels present there [128,129]. Lu et al. designed innovative T_2_/T_1_ switchable CAs by modifying IONPs with i-motif DNAs, as shown in Figure 8b [126]. Their findings demonstrated that the acidic environment of tumors triggers the disassembly of the responsive agents into well-dispersed IONPs, leading to a significant reduction in transverse relaxation time and facilitating the shift of MRI modularity from T_2_ enhancement to T_1_ enhancement.

Li et al. present pH-sensitive iron oxide nanoparticle assemblies (IONAs) that are cross-linked with small-molecule aldehyde derivative ligands [127], as shown in Figure 8c. The reversible formation and cleavage of hydrazone linkages in neutral and acidic conditions allow the nanoassemblies to adapt to pH fluctuations. At neutral pH, the IONAs are structurally stable due to the robust hydrazone bonds. In contrast, in the acidic tumor microenvironment, these bonds are cleaved, resulting in the rapid breakdown of IONAs into many hydrophilic, ultra-small IONPs. This process significantly enhances T_1_ MR contrast, as evidenced by r_1_ value assessments across different pH levels.

### 6.2. Distance Adjustment

**Figure 9:**
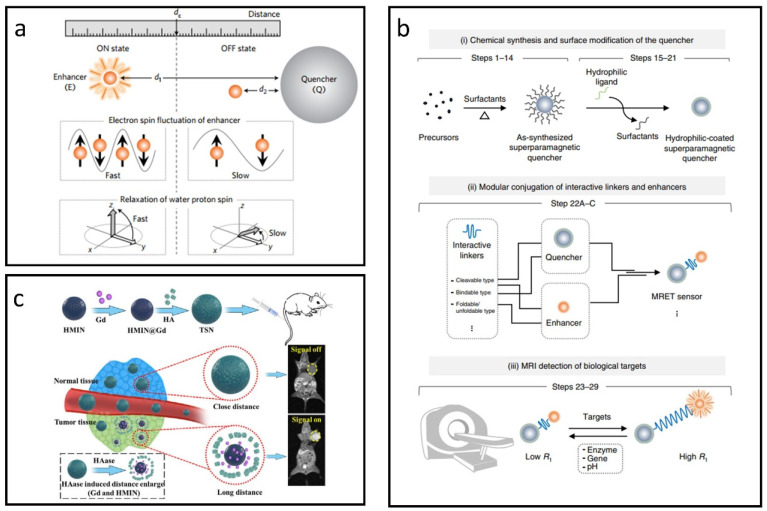
Approaches for designing IONP-based responsive CAs utilizing the MRET effect. (**a**) Schematic diagram illustrating distance-dependent MRET. Reproduced with permission [130]. Copyright 2017, Nature Publishing Group. (**b**) Overview of the experimental workflow for developing MRET-based responsive CAs. Reproduced with permission [131]. Copyright 2018, Nature Publishing Group. (**c**) HAase-triggered responsive CAs leveraging the MRET effect. Reproduced with permission [132]. Copyright 2021, Elsevier Ltd.

Recent studies have demonstrated that superparamagnetic nanoparticles can significantly reduce the T_1_ relaxivity of paramagnetic CAs, leading to new responsive CA concepts. A classic magnetic resonance tuning (MRET) system utilizes the interaction between superparamagnetic IONPs and paramagnetic Gd chelates, allowing the T_1_ signal to be toggled “on” or “off” based on the distance between them [130]. As shown in Figure 9a, when the enhancer moves away from the quencher, it promotes water proton relaxation, resulting in a stronger T_1_ signal (“on” state). Conversely, close proximity between the two inhibits effective water proton relaxation, producing a weaker T_1_ signal (“off” state).

Utilizing the MRET mechanism, a comprehensive protocol has been developed for creating responsive CAs that can non-invasively identify biologically significant targets using MRI [131]. As illustrated in Figure 9b, this protocol involves three main steps: (i) chemical synthesis and surface modification of the quencher, (ii) conjugation with interactive linkers and enhancers, and (iii) MRI detection of biological targets.

For now, MRET-based CAs have been widely used in many fields [132,133,134,135]. Liu et al. employed superparamagnetic hollow mesoporous IONPs to encapsulate the T_1_ CA (Gd), and subsequently obstructed the mesopores using biocompatible hyaluronic acid (HA), see Figure 9c [132]. In healthy tissues with low levels of hyaluronidase (HAase), Gd remained trapped within the IONPs, maintaining close proximity between them and effectively turning the T_1_ signal “off” due to magnetic interference. In contrast, in tumor environments with elevated HAase levels, Gd was quickly released from the IONPs, increasing the separation between them. This reduction in interference enabled the T_1_ signal to be activated, allowing for tumor-targeted MR imaging through a distance-dependent mechanism.

Gao et al. created an FAPα-responsive CA to quantitatively assess liver fibrosis [133]. This CA is composed of superparamagnetic amorphous iron nanoparticles (AFeNPs) and paramagnetic gadoteric acid (Gd-DOTA), connected through FAPα-responsive peptide chains (ASGPAGPAs). As liver fibrosis worsens, higher levels of FAPα lead to the cleavage of more ASGPAGPAs, which in turn boosts the T_1_-MRI signal from Gd-DOTA. Conversely, the signal stays reduced because of the distance-dependent MRET effect between AFeNPs and Gd-DOTA. The CA effectively distinguishes between F1, F2, F3, and F4 fibrosis, with area under the curve values recorded at 99.8%, 66.7%, 70.4%, and 96.3% from patient samples, respectively.

In contrast to the MRET system that features T_1_ CAs positioned externally to T_2_ CAs, there is a growing trend of research exploring the scenario where T_1_ CAs are integrated within T_2_ CAs. One approach involves doping superparamagnetic IONPs with paramagnetic T_1_ CAs. Zhou et al. found that embedding Gd compounds (such as Gd_2_O_3_) within superparamagnetic IONPs enhances local magnetic field strengths when exposed to an external magnetic field, leading to a synergistic increase in both r_1_ and r_2_ relaxivity values [136]. The Gd-doped IONPs displayed a higher r_2_ (146.5 mM^−1^·s^−1^) compared to Fe_3_O_4_ (125.4 mM^−1^·s^−1^) of similar dimensions, and a higher r_1_ (69.5 mM^−1^·s^−1^) than Gd_2_O_3_ (12.1 mM^−1^·s^−1^) of comparable size. Moreover, Gd_2_O_3_ nanoparticles did not exhibit enhanced T_2_ contrast, whereas Fe_3_O_4_ nanoparticles showed limited improvements in T_1_ contrast. This synergistic enhancement of r_1_ and r_2_ has also been observed in Mn- and Eu-doped IONP systems, suggesting potential strategies for developing dual-mode T_1_–T_2_ MRI CAs [137].

## 7. The Biocompatibility, Organ Accumulation, and Routes of Clearance of IONPs

IONPs are primarily biodegradable, with their degradation resembling ferritin metabolism, where lysosomal enzymes break them down to release iron ions [138]. Studies indicate that IONPs typically exhibit low toxicity, resulting in only temporary effects such as oxidative stress, without causing long-term organ damage [139]. Significant adverse effects were noted only at very high doses (500 mg Fe/kg). For MRI applications, intravenous injection is the most common method for administering IONPs. These nanoparticles are mainly absorbed by the liver and spleen, which play a crucial role in their elimination from the body, forming part of the mononuclear phagocytic system (MPS). While often used interchangeably, it is important to note that RES specifically includes sinusoidal epithelial cells of the liver, whereas MPS encompasses circulating monocytes and macrophages in various organs [140]. Specialized macrophages such as Kupffer cells in the liver and microglial cells in the brain help clear pathogens and foreign materials, including IONPs, through phagocytosis [141]. Excessive amounts of IONPs can accumulate in other tissues, like the lungs and heart, particularly after high-dose injections. Prior to uptake by the liver and spleen, IONPs undergo opsonization, where specific proteins attach to their surfaces, facilitating recognition by macrophages.

Biodistribution is also closely linked to the size of IONPs. Research indicates that the hydrodynamic diameter (DH) of IONPs significantly affects their pharmacokinetics and distribution in various organs [142]. Larger IONPs are absorbed more rapidly by the liver and spleen and have shorter circulation times, while smaller IONPs primarily accumulate in lymph nodes with longer circulation times [143]. In a study comparing Ferumoxtran-10 and Ferumoxides, 30 nm Ferumoxtran-10 exhibited significant localization in the spleen and liver, while 80 nm Ferumoxides primarily accumulated in the liver [144]. Another investigation of PEG-coated magnetic nanoparticles showed that both types mainly distributed in the liver and spleen, with lower levels found in the lungs, heart, and kidneys [145]. Factors like charge and morphology also play a role. For instance, in vivo studies of carboxyl-coated IONPs showed that smaller particles (10 nm) had the highest liver uptake, while larger ones (40 nm) accumulated more in the spleen [146]. One study on magnetic mesoporous silica nanoparticles (M-MSNPs) found that nanorods accumulated more in the spleen than spheres, which were mainly located in the liver [147]. This difference was attributed to the larger surface area of nanorods, resulting in slower clearance rates and greater accumulation in target tissues.

As discussed above, the liver and kidney play an important role in eliminating IONPs; thus, the consequence of IONPs in patients with kidney and liver dysfunctions is worth investigation. Recently, IONPs have been utilized in diagnosing focal liver lesions and assessing fibrosis progression in steatohepatitis by measuring their levels in Kupffer cells [148]. In vitro studies indicate that the cytotoxicity of IONPs varies based on cell type, nanoparticle size, and coating materials. Elevated iron levels in liver tissues have been consistently observed, although this iron overload does not significantly impair liver function or provoke major immunotoxic responses in healthy models. Nonetheless, excess iron raises safety concerns due to its potential to induce oxidative stress and increase lipid peroxidation. These concerns are exacerbated by clinical manifestations such as cirrhosis in liver cancer, characterized by fibrosis and reduced liver function. In fibrosis, lipid peroxidation from excess iron poses a risk in non-alcoholic fatty liver disease, as the liver is crucial for lipid metabolism. Thus, IONP-induced iron overload could potentially increase the risk of progression in cirrhosis patients, particularly in the context of the necroinflammatory environment in the liver.

Unlike liver studies, the effects of IONPs in individuals with kidney disease are less documented. Approved in 2009 and 2012 for treating iron deficiency in chronic kidney disease under the brand names Ferumoxytol (or Feraheme) and Rienso, IONPs have shown high dose tolerance up to 510 mg and elevated hemoglobin levels [149]. Recent studies reported minor side effects in a small number of patients and no adverse events in kidney disease patients. However, a larger observational study revealed rare but serious adverse effects, and common side effects were reported in European clinical studies. Despite these findings, the long-term safety of IONPs remains not fully understood.

## 8. Conclusions and Outlook

IONPs, traditionally used as T_2_-MRI CAs, have recently surfaced as a promising nontoxic substitute for gadolinium-based contrast agents (GBCAs) in T_1_ imaging. This review delves into key aspects of IONPs as T_1_ contrast agents, an area that has attracted considerable interest in recent years. Several effective synthetic methods have been utilized to produce these nanoparticles, including chemical precipitation, solvothermal techniques, thermal decomposition, and microemulsion techniques. Among these methods, thermal decomposition stands out for its uniformity and precise reaction control, while chemical precipitation is more suitable for large-scale production. The SBM theory and quantum mechanical outer sphere theory are fundamental to the design of IONP-based CAs. To improve T_1_ contrast effectiveness, it is crucial to decrease r_2_ and increase r_1_ for IONPs. Important factors affecting T_1_ performance include nanoparticle size, morphology, surface modifications, valence states, and oxygen vacancies. Additionally, we introduced strategies for designing IONP-based responsive CAs, such as self-assembly/disassembly and magnetic resonance tuning effect.

In comparison to well-characterized Gd-based T_1_ CAs and larger IONPs T_2_ CAs, the development of IONP-based T_1_ CAs is still in its infancy. Translating these advancements to clinical use involves practical challenges like long-term storage, biological interactions, circulation duration, large-scale synthesis, and cost, which may hinder the implementation of the proposed strategies. For instance, while reducing particle size can lower r_2_ and enhance r_1_, the high surface energy can result in aggregation, complicating effective steric stabilization. Further studies on the in vitro and in vivo toxicity of doped metal ions are also required. Detailed information regarding biocompatibility, biodistribution, pharmacokinetics, and long-term outcomes is vital for progressing clinical applications. A careful evaluation of all relevant parameters is necessary for IONP-based T_1_ CAs to move beyond the laboratory.

## References

[1] Iyad N., Ahmad M.S., Alkhatib S.G., Hjouj M. (2023). Gadolinium contrast agents-challenges and opportunities of a multidisciplinary approach: Literature review. Eur. J. Radiol. Open.

[2] Kastelik-Hryniewiecka A., Jewula P., Bakalorz K., Kramer-Marek G., Kuźnik N. (2022). Targeted PET/MRI Imaging Super Probes: A Critical Review of Opportunities and Challenges. Int. J. Nanomed..

[3] Ni D., Bu W., Ehlerding E.B., Cai W., Shi J. (2017). Engineering of inorganic nanoparticles as magnetic resonance imaging contrast agents. Chem. Soc. Rev..

[4] Babić-Stojić B., Jokanović V., Milivojević D., Požek M., Jagličić Z., Makovec D., Orsini N.J., Marković M., Arsikin K., Paunović V. (2018). Ultrasmall iron oxide nanoparticles: Magnetic and NMR relaxometric properties. Curr. Appl. Phys..

[5] Peng Y.-K., Tsang S.C.E., Chou P.-T. (2016). Chemical design of nanoprobes for T1-weighted magnetic resonance imaging. Mater. Today.

[6] Estelrich J., Sánchez-Martín M.J., Busquets M.A. (2015). Nanoparticles in magnetic resonance imaging: From simple to dual contrast agents. Int. J. Nanomed..

[7] Yan X., Li S., Yan H., Yu C., Liu F. (2023). IONPs-Based Medical Imaging in Cancer Care: Moving Beyond Traditional Diagnosis and Therapeutic Assessment. Int. J. Nanomed..

[8] Boldrini L., Alongi F., Romano A., Charles Davies D., Bassetti M., Chiloiro G., Corradini S., Gambacorta M.A., Placidi L., Tree A.C. (2023). Current practices and perspectives on the integration of contrast agents in MRI-guided radiation therapy clinical practice: A worldwide survey. Clin. Transl. Radiat. Oncol..

[9] Wang H., Revia R., Wang K., Kant R.J., Mu Q., Gai Z., Hong K., Zhang M. (2017). Paramagnetic Properties of Metal-Free Boron-Doped Graphene Quantum Dots and Their Application for Safe Magnetic Resonance Imaging. Adv. Mater..

[10] Kanda T., Ishii K., Kawaguchi H., Kitajima K., Takenaka D. (2013). High Signal Intensity in the Dentate Nucleus and Globus Pallidus on Unenhanced T1-weighted MR Images: Relationship with Increasing Cumulative Dose of a Gadolinium-based Contrast Material. Radiology.

[11] Ramalho J., Semelka R.C., Ramalho M., Nunes R.H., AlObaidy M., Castillo M. (2016). Gadolinium-Based Contrast Agent Accumulation and Toxicity: An Update. Am. J. Neuroradiol..

[12] Bao Y., Sherwood J.A., Sun Z. (2018). Magnetic iron oxide nanoparticles as T1 contrast agents for magnetic resonance imaging. J. Mater. Chem. C.

[13] Pourmadadi M., Rahmani E., Shamsabadipour A., Mahtabian S., Ahmadi M., Rahdar A., Díez-Pascual A.M. (2022). Role of Iron Oxide (Fe_2_O_3_) Nanocomposites in Advanced Biomedical Applications: A State-of-the-Art Review. Nanomaterials.

[14] Xie M., Meng F., Wang P., Díaz-García A.M., Parkhats M., Santos-Oliveira R., Asim M.H., Bostan N., Gu H., Yang L. (2024). Surface Engineering of Magnetic Iron Oxide Nanoparticles for Breast Cancer Diagnostics and Drug Delivery. Int. J. Nanomed..

[15] Yang Y., Liu Y., Song L., Cui X., Zhou J., Jin G., Boccaccini A.R., Virtanen S. (2023). Iron oxide nanoparticle-based nanocomposites in biomedical application. Trends Biotechnol..

[16] Wang S., He H., Mao Y., Zhang Y., Gu N. (2024). Advances in Atherosclerosis Theranostics Harnessing Iron Oxide-Based Nanoparticles. Adv. Sci..

[17] Jeon M., Halbert M.V., Stephen Z.R., Zhang M. (2021). Iron Oxide Nanoparticles as T1 Contrast Agents for Magnetic Resonance Imaging: Fundamentals, Challenges, Applications, and Prospectives. Adv. Mater..

[18] Zhao Z., Li M., Zeng J., Huo L., Liu K., Wei R., Ni K., Gao J. (2022). Recent advances in engineering iron oxide nanoparticles for effective magnetic resonance imaging. Bioact. Mater..

[19] Yang J., Feng J., Yang S., Xu Y., Shen Z. (2023). Exceedingly Small Magnetic Iron Oxide Nanoparticles for T1-Weighted Magnetic Resonance Imaging and Imaging-Guided Therapy of Tumors. Small.

[20] Penfield J.G., Reilly R.F. (2007). What nephrologists need to know about gadolinium. Nat. Clin. Pract. Nephrol..

[21] Perez-Rodriguez J., Lai S., Ehst B.D., Fine D.M., Bluemke D.A. (2009). Nephrogenic Systemic Fibrosis: Incidence, Associations, and Effect of Risk Factor Assessment—Report of 33 Cases. Radiology.

[22] Li B., Xie G., Zou Q., Zhao Y., Han B., Yu C., Pan J., Sun S.-K. (2023). Non-invasive Diagnosis and Postoperative Evaluation of Carotid Artery Stenosis by BSA-Gd2O3 Nanoparticles-Based Magnetic Resonance Angiography. ACS Appl. Bio Mater..

[23] Jiang Z., Xia B., Ren F., Bao B., Xing W., He T., Li Z. (2022). Boosting Vascular Imaging-Performance and Systemic Biosafety of Ultra-Small NaGdF4 Nanoparticles via Surface Engineering with Rationally Designed Novel Hydrophilic Block Co-Polymer. Small Methods.

[24] Youk J.H., Lee J.M., Kim C.S. (2004). MRI for Detection of Hepatocellular Carcinoma: Comparison of Mangafodipir Trisodium and Gadopentetate Dimeglumine Contrast Agents. Am. J. Roentgenol..

[25] Elizondo G., Fretz C.J., Stark D.D., Rocklage S.M., Quay S.C., Worah D., Tsang Y.M., Chen M.C., Ferrucci J.T. (1991). Preclinical evaluation of MnDPDP: New paramagnetic hepatobiliary contrast agent for MR imaging. Radiology.

[26] Chen Y., Yin Q., Ji X., Zhang S., Chen H., Zheng Y., Sun Y., Qu H., Wang Z., Li Y. (2012). Manganese oxide-based multifunctionalized mesoporous silica nanoparticles for pH-responsive MRI, ultrasonography and circumvention of MDR in cancer cells. Biomaterials.

[27] Yuan D., Ding L., Sun Z., Li X. (2018). MRI/Fluorescence bimodal amplification system for cellular GSH detection and tumor cell imaging based on manganese dioxide nanosheet. Sci. Rep..

[28] Kuźnik N., Wyskocka M. (2016). Iron(III) Contrast Agent Candidates for MRI: A Survey of the Structure–Effect Relationship in the Last 15 Years of Studies. Eur. J. Inorg. Chem..

[29] Xie J., Haeckel A., Hauptmann R., Ray I.P., Limberg C., Kulak N., Hamm B., Schellenberger E. (2021). Iron(III)-tCDTA derivatives as MRI contrast agents: Increased T1 relaxivities at higher magnetic field strength and pH sensing. Magn. Reson. Med..

[30] Wang Y.X. (2015). Current status of superparamagnetic iron oxide contrast agents for liver magnetic resonance imaging. World J. Gastroenterol..

[31] Whitlow J., Pacelli S., Paul A. (2017). Multifunctional nanodiamonds in regenerative medicine: Recent advances and future directions. J. Control. Release.

[32] Kaur R., Badea I. (2013). Nanodiamonds as novel nanomaterials for biomedical applications: Drug delivery and imaging systems. Int. J. Nanomed..

[33] Terada D., Genjo T., Segawa T.F., Igarashi R., Shirakawa M. (2020). Nanodiamonds for bioapplications–specific targeting strategies. Biochim. Biophys. Acta (BBA)-Gen. Subj..

[34] Chipaux M., van der Laan K.J., Hemelaar S.R., Hasani M., Zheng T., Schirhagl R. (2018). Nanodiamonds and Their Applications in Cells. Small.

[35] Manus L.M., Mastarone D.J., Waters E.A., Zhang X.-Q., Schultz-Sikma E.A., MacRenaris K.W., Ho D., Meade T.J. (2010). Gd(III)-Nanodiamond Conjugates for MRI Contrast Enhancement. Nano Lett..

[36] Lazovic J., Goering E., Wild A.-M., Schützendübe P., Shiva A., Löffler J., Winter G., Sitti M. (2024). Nanodiamond-Enhanced Magnetic Resonance Imaging. Adv. Mater..

[37] Trifoi A.R., Matei E., Râpă M., Berbecaru A.-C., Panaitescu C., Banu I., Doukeh R. (2023). Coprecipitation nanoarchitectonics for the synthesis of magnetite: A review of mechanism and characterization. React. Kinet. Mech. Catal..

[38] Ahn T., Kim J.H., Yang H.-M., Lee J.W., Kim J.-D. (2012). Formation Pathways of Magnetite Nanoparticles by Coprecipitation Method. J. Phys. Chem. C.

[39] Vreeland E.C., Watt J., Schober G.B., Hance B.G., Austin M.J., Price A.D., Fellows B.D., Monson T.C., Hudak N.S., Maldonado-Camargo L. (2015). Enhanced Nanoparticle Size Control by Extending LaMer’s Mechanism. Chem. Mater..

[40] Chen B., Guo Z., Guo C., Mao Y., Qin Z., Ye D., Zang F., Lou Z., Zhang Z., Li M. (2020). Moderate cooling coprecipitation for extremely small iron oxide as a pH dependent T1-MRI contrast agent. Nanoscale.

[41] Mao Y., Li Y., Zang F., Yu H., Yan S., Song Q., Qin Z., Sun J., Chen B., Huang X. (2022). Continuous synthesis of extremely small-sized iron oxide nanoparticles used for T1-weighted magnetic resonance imaging via a fluidic reactor. Sci. China Mater..

[42] Chen B., Sun J., Fan F., Zhang X., Qin Z., Wang P., Li Y., Zhang X., Liu F., Liu Y. (2018). Ferumoxytol of ultrahigh magnetization produced by hydrocooling and magnetically internal heating co-precipitation. Nanoscale.

[43] Arias L.S., Pessan J.P., Vieira A.P., Lima T.M., Delbem A.C., Monteiro D.R. (2018). Iron Oxide Nanoparticles for Biomedical Applications: A Perspective on Synthesis, Drugs, Antimicrobial Activity, and Toxicity. Antibiotics.

[44] Joseyphus R.J., Shinoda K., Kodama D., Jeyadevan B. (2010). Size controlled Fe nanoparticles through polyol process and their magnetic properties. Mater. Chem. Phys..

[45] Zheng Y.-Y., Sun Q., Duan Y.-H., Zhai J., Zhang L.-L., Wang J.-X. (2020). Controllable synthesis of monodispersed iron oxide nanoparticles by an oxidation-precipitation combined with solvothermal process. Mater. Chem. Phys..

[46] Luo Y., Yang J., Yan Y., Li J., Shen M., Zhang G., Mignani S., Shi X. (2015). RGD-functionalized ultrasmall iron oxide nanoparticles for targeted T1-weighted MR imaging of gliomas. Nanoscale.

[47] Nozawa R., Naka T., Kurihara M., Togashi T. (2021). Size-tunable synthesis of iron oxide nanocrystals by continuous seed-mediated growth: Role of alkylamine species in the stepwise thermal decomposition of iron(ii) oxalate. Dalton Trans..

[48] Vangijzegem T., Lecomte V., Ternad I., Van Leuven L., Muller R.N., Stanicki D., Laurent S. (2023). Superparamagnetic Iron Oxide Nanoparticles (SPION): From Fundamentals to State-of-the-Art Innovative Applications for Cancer Therapy. Pharmaceutics.

[49] Andrade R.G.D., Veloso S.R.S., Castanheira E.M.S. (2020). Shape Anisotropic Iron Oxide-Based Magnetic Nanoparticles: Synthesis and Biomedical Applications. Int. J. Mol. Sci..

[50] Hufschmid R., Arami H., Ferguson R.M., Gonzales M., Teeman E., Brush L.N., Browning N.D., Krishnan K.M. (2015). Synthesis of phase-pure and monodisperse iron oxide nanoparticles by thermal decomposition. Nanoscale.

[51] Fokina V., Wilke M., Dulle M., Ehlert S., Förster S. (2022). Size Control of Iron Oxide Nanoparticles Synthesized by Thermal Decomposition Methods. J. Phys. Chem. C.

[52] Kim B.H., Lee N., Kim H., An K., Park Y.I., Choi Y., Shin K., Lee Y., Kwon S.G., Na H.B. (2011). Large-Scale Synthesis of Uniform and Extremely Small-Sized Iron Oxide Nanoparticles for High-Resolution T1 Magnetic Resonance Imaging Contrast Agents. J. Am. Chem. Soc..

[53] Assa F., Jafarizadeh-Malmiri H., Ajamein H., Anarjan N., Vaghari H., Sayyar Z., Berenjian A. (2016). A biotechnological perspective on the application of iron oxide nanoparticles. Nano Res..

[54] Salvador M., Gutiérrez G., Noriega S., Moyano A., Blanco-López M.C., Matos M. (2021). Microemulsion Synthesis of Superparamagnetic Nanoparticles for Bioapplications. Int. J. Mol. Sci..

[55] Ali A., Zafar H., Zia M., ul Haq I., Phull A.R., Ali J.S., Hussain A. (2016). Synthesis, characterization, applications, and challenges of iron oxide nanoparticles. Nanotechnol. Sci. Appl..

[56] Koutzarova T., Kolev S., Ghelev C., Paneva D., Nedkov I. (2006). Microstructural study and size control of iron oxide nanoparticles produced by microemulsion technique. Phys. Status Solidi C.

[57] Okoli C., Sanchez-Dominguez M., Boutonnet M., Järås S., Civera C., Solans C., Kuttuva G.R. (2012). Comparison and Functionalization Study of Microemulsion-Prepared Magnetic Iron Oxide Nanoparticles. Langmuir.

[58] Drmota A., Drofenik M., Koselj J., Žnidaršič A., Reza N. (2012). Microemulsion Method for Synthesis of Magnetic Oxide Nanoparticles. Microemulsions.

[59] Liu W., Yin S., Hu Y., Deng T., Li J. (2021). Microemulsion-Confined Biomineralization of PEGylated Ultrasmall Fe_3_O_4_ Nanocrystals for T2-T1 Switchable MRI of Tumors. Anal. Chem..

[60] Soenen S.J.H., De Cuyper M. (2009). Assessing cytotoxicity of (iron oxide-based) nanoparticles: An overview of different methods exemplified with cationic magnetoliposomes. Contrast Media Mol. Imaging.

[61] Pedro T., María del Puerto M., Sabino V.-V., Teresita G.-C., Carlos J.S. (2003). The preparation of magnetic nanoparticles for applications in biomedicine. J. Phys. D Appl. Phys..

[62] Stephen Z.R., Kievit F.M., Zhang M. (2011). Magnetite nanoparticles for medical MR imaging. Mater. Today.

[63] Hanson L.G. (2008). Is quantum mechanics necessary for understanding magnetic resonance?. Concepts Magn. Reson. Part A.

[64] Tromsdorf U.I., Bigall N.C., Kaul M.G., Bruns O.T., Nikolic M.S., Mollwitz B., Sperling R.A., Reimer R., Hohenberg H., Parak W.J. (2007). Size and Surface Effects on the MRI Relaxivity of Manganese Ferrite Nanoparticle Contrast Agents. Nano Lett..

[65] Wei H., Bruns O.T., Kaul M.G., Hansen E.C., Barch M., Wiśniowska A., Chen O., Chen Y., Li N., Okada S. (2017). Exceedingly small iron oxide nanoparticles as positive MRI contrast agents. Proc. Natl. Acad. Sci. USA.

[66] Xue S., Qiao J., Jiang J., Hubbard K., White N., Wei L., Li S., Liu Z.-R., Yang J.J. (2014). Design of ProCAs (Protein-Based Gd^3+^ MRI Contrast Agents) with High Dose Efficiency and Capability for Molecular Imaging of Cancer Biomarkers. Med. Res. Rev..

[67] Smeraldo A., Netti P.A., Torino E. (2020). New Strategies in the Design of Paramagnetic CAs. Contrast Media Mol. Imaging.

[68] Lacerda S., Ndiaye D., Tóth É., Hubbard C.D., van Eldik R. (2021). Chapter Four—MRI relaxation agents based on transition metals. Advances in Inorganic Chemistry.

[69] Zairov R., Pizzanelli S., Dovzhenko A.P., Nizameev I., Orekhov A., Arkharova N., Podyachev S.N., Sudakova S., Mustafina A.R., Calucci L. (2020). Paramagnetic Relaxation Enhancement in Hydrophilic Colloids Based on Gd(III) Complexes with Tetrathia- and Calix[4]arenes. J. Phys. Chem. C.

[70] Bonnet C.S., Fries P.H., Crouzy S., Delangle P. (2010). Outer-Sphere Investigation of MRI Relaxation Contrast Agents. Example of a Cyclodecapeptide Gadolinium Complex with Second-Sphere Water. J. Phys. Chem. B.

[71] Botta M. (2000). Second Coordination Sphere Water Molecules and Relaxivity of Gadolinium(III) Complexes: Implications for MRI Contrast Agents. Eur. J. Inorg. Chem..

[72] Zhang J., Dai L., He L., Bhattarai A., Chan C.-M., Tai W.C.-S., Vardhanabhuti V., Law G.-L. (2023). Design and synthesis of chiral DOTA-based MRI contrast agents with remarkable relaxivities. Commun. Chem..

[73] Wu Y., Zhao S., Xu Y., Tang C., Feng Y., Zhang M., Yang H., Ma Y., Li Y., Wang W. (2024). A Hexanuclear Gadolinium(III)-Based Nanoprobe for Magnetic Resonance Imaging of Tumor Apoptosis. ACS Appl. Nano Mater..

[74] Solomon I., Bloembergen N. (1956). Nuclear Magnetic Interactions in the HF Molecule. J. Chem. Phys..

[75] Freed J.H. (1978). Dynamic effects of pair correlation functions on spin relaxation by translational diffusion in liquids. II. Finite jumps and independent T1 processes. J. Chem. Phys..

[76] Laurent S., Forge D., Port M., Roch A., Robic C., Vander Elst L., Muller R.N. (2008). Magnetic Iron Oxide Nanoparticles: Synthesis, Stabilization, Vectorization, Physicochemical Characterizations, and Biological Applications. Chem. Rev..

[77] Zhang W., Liu L., Chen H., Hu K., Delahunty I., Gao S., Xie J. (2018). Surface impact on nanoparticle-based magnetic resonance imaging contrast agents. Theranostics.

[78] Jacques V., Dumas S., Sun W.-C., Troughton J.S., Greenfield M.T., Caravan P. (2010). High-Relaxivity Magnetic Resonance Imaging Contrast Agents Part 2: Optimization of Inner- and Second-Sphere Relaxivity. Investig. Radiol..

[79] Powell D.H., Dhubhghaill O.M.N., Pubanz D., Helm L., Lebedev Y.S., Schlaepfer W., Merbach A.E. (1996). Structural and Dynamic Parameters Obtained from 17O NMR, EPR, and NMRD Studies of Monomeric and Dimeric Gd^3+^ Complexes of Interest in Magnetic Resonance Imaging:  An Integrated and Theoretically Self-Consistent Approach1. J. Am. Chem. Soc..

[80] Peng E., Wang F., Xue J.M. (2015). Nanostructured magnetic nanocomposites as MRI contrast agents. J. Mater. Chem. B.

[81] Zhou Z., Yang L., Gao J., Chen X. (2019). Structure–Relaxivity Relationships of Magnetic Nanoparticles for Magnetic Resonance Imaging. Adv. Mater..

[82] Liu M., Yuan J., Wang G., Ni N., Lv Q., Liu S., Gong Y., Zhao X., Wang X., Sun X. (2023). Shape programmable T1–T2 dual-mode MRI nanoprobes for cancer theranostics. Nanoscale.

[83] Farinha P., Coelho J.M.P., Reis C.P., Gaspar M.M. (2021). A Comprehensive Updated Review on Magnetic Nanoparticles in Diagnostics. Nanomaterials.

[84] Kostopoulou A., Lappas A. (2015). Colloidal magnetic nanocrystal clusters: Variable length-scale interaction mechanisms, synergetic functionalities and technological advantages. Nanotechnol. Rev..

[85] Xu F., Cheng C., Chen D.-X., Gu H. (2012). Magnetite Nanocrystal Clusters with Ultra-High Sensitivity in Magnetic Resonance Imaging. ChemPhysChem.

[86] Yablonskiy D.A., Haacke E.M. (1994). Theory of NMR signal behavior in magnetically inhomogeneous tissues: The static dephasing regime. Magn. Reson. Med..

[87] Brooks R.A. (2002). T2-shortening by strongly magnetized spheres: A chemical exchange model. Magn. Reson. Med..

[88] Balasubramaniam S., Kayandan S., Lin Y.-N., Kelly D.F., House M.J., Woodward R.C., St. Pierre T.G., Riffle J.S., Davis R.M. (2014). Toward Design of Magnetic Nanoparticle Clusters Stabilized by Biocompatible Diblock Copolymers for T2-Weighted MRI Contrast. Langmuir.

[89] Carroll M.R.J., Woodward R.C., House M.J., Teoh W.Y., Amal R., Hanley T.L., St Pierre T.G. (2010). Experimental validation of proton transverse relaxivity models for superparamagnetic nanoparticle MRI contrast agents. Nanotechnology.

[90] Zhao S., Yu X., Qian Y., Chen W., Shen J. (2020). Multifunctional magnetic iron oxide nanoparticles: An advanced platform for cancer theranostics. Theranostics.

[91] Macher T., Totenhagen J., Sherwood J., Qin Y., Gurler D., Bolding M.S., Bao Y. (2015). Ultrathin Iron Oxide Nanowhiskers as Positive Contrast Agents for Magnetic Resonance Imaging. Adv. Funct. Mater..

[92] Wu M., Meng Q., Chen Y., Xu P., Zhang S., Li Y., Zhang L., Wang M., Yao H., Shi J. (2014). Ultrasmall Confined Iron Oxide Nanoparticle MSNs as a pH-Responsive Theranostic Platform. Adv. Funct. Mater..

[93] Morales M.P., Veintemillas-Verdaguer S., Montero M.I., Serna C.J., Roig A., Casas L., Martínez B., Sandiumenge F. (1999). Surface and Internal Spin Canting in γ-Fe_2_O_3_ Nanoparticles. Chem. Mater..

[94] Baaziz W., Pichon B.P., Fleutot S., Liu Y., Lefevre C., Greneche J.-M., Toumi M., Mhiri T., Begin-Colin S. (2014). Magnetic Iron Oxide Nanoparticles: Reproducible Tuning of the Size and Nanosized-Dependent Composition, Defects, and Spin Canting. J. Phys. Chem. C.

[95] Shen Z., Chen T., Ma X., Ren W., Zhou Z., Zhu G., Zhang A., Liu Y., Song J., Li Z. (2017). Multifunctional Theranostic Nanoparticles Based on Exceedingly Small Magnetic Iron Oxide Nanoparticles for T1-Weighted Magnetic Resonance Imaging and Chemotherapy. ACS Nano.

[96] Johnson N.J.J., Oakden W., Stanisz G.J., Scott Prosser R., van Veggel F.C.J.M. (2011). Size-Tunable, Ultrasmall NaGdF_4_ Nanoparticles: Insights into Their T1 MRI Contrast Enhancement. Chem. Mater..

[97] Yang L., Wang Z., Ma L., Li A., Xin J., Wei R., Lin H., Wang R., Chen Z., Gao J. (2018). The Roles of Morphology on the Relaxation Rates of Magnetic Nanoparticles. ACS Nano.

[98] Zhou Z., Zhao Z., Zhang H., Wang Z., Chen X., Wang R., Chen Z., Gao J. (2014). Interplay between Longitudinal and Transverse Contrasts in Fe_3_O_4_ Nanoplates with (111) Exposed Surfaces. ACS Nano.

[99] Noh S.-h., Na W., Jang J.-t., Lee J.-H., Lee E.J., Moon S.H., Lim Y., Shin J.-S., Cheon J. (2012). Nanoscale Magnetism Control via Surface and Exchange Anisotropy for Optimized Ferrimagnetic Hysteresis. Nano Lett..

[100] Schladt T.D., Schneider K., Schild H., Tremel W. (2011). Synthesis and bio-functionalization of magnetic nanoparticles for medical diagnosis and treatment. Dalton Trans..

[101] Wang J., Jia Y., Wang Q., Liang Z., Han G., Wang Z., Lee J., Zhao M., Li F., Bai R. (2021). An Ultrahigh-Field-Tailored T1–T2 Dual-Mode MRI Contrast Agent for High-Performance Vascular Imaging. Adv. Mater..

[102] LaConte L.E.W., Nitin N., Zurkiya O., Caruntu D., O’Connor C.J., Hu X., Bao G. (2007). Coating thickness of magnetic iron oxide nanoparticles affects R2 relaxivity. J. Magn. Reson. Imaging.

[103] Zeng J., Jing L., Hou Y., Jiao M., Qiao R., Jia Q., Liu C., Fang F., Lei H., Gao M. (2014). Anchoring Group Effects of Surface Ligands on Magnetic Properties of Fe_3_O_4_ Nanoparticles: Towards High Performance MRI Contrast Agents. Adv. Mater..

[104] Smolensky E.D., Park H.-Y.E., Berquó T.S., Pierre V.C. (2011). Surface functionalization of magnetic iron oxide nanoparticles for MRI applications—Effect of anchoring group and ligand exchange protocol. Contrast Media Mol. Imaging.

[105] Palma S.I.C.J., Marciello M., Carvalho A., Veintemillas-Verdaguer S., Morales M.d.P., Roque A.C.A. (2015). Effects of phase transfer ligands on monodisperse iron oxide magnetic nanoparticles. J. Colloid Interface Sci..

[106] Bertini I., Capozzi F., Luchinat C., Xia Z. (1993). Nuclear and electron relaxation of hexaaquairon(3+). J. Phys. Chem..

[107] Ducommun Y., Newman K.E., Merbach A.E. (1980). High-pressure oxygen-17 NMR evidence for a gradual mechanistic changeover from Ia to Id for water exchange on divalent octahedral metal ions going from manganese(II) to nickel(II). Inorg. Chem..

[108] La Mar G.N., Walker F.A. (1973). Proton nuclear magnetic resonance and electron spin resonance investigation of the electronic structure and magnetic properties of synthetic low-spin ferric porphyrins. J. Am. Chem. Soc..

[109] Zhao Z., Xu K., Fu C., Liu H., Lei M., Bao J., Fu A., Yu Y., Zhang W. (2019). Interfacial engineered gadolinium oxide nanoparticles for magnetic resonance imaging guided microenvironment-mediated synergetic chemodynamic/photothermal therapy. Biomaterials.

[110] Zhao Z., Sun C., Bao J., Yang L., Wei R., Cheng J., Lin H., Gao J. (2018). Surface manganese substitution in magnetite nanocrystals enhances T1 contrast ability by increasing electron spin relaxation. J. Mater. Chem. B.

[111] Park J.C., Lee G.T., Kim H.-K., Sung B., Lee Y., Kim M., Chang Y., Seo J.H. (2018). Surface Design of Eu-Doped Iron Oxide Nanoparticles for Tuning the Magnetic Relaxivity. ACS Appl. Mater. Interfaces.

[112] Han G., Deng Y., Sun J., Ling J., Shen Z. (2015). Research into europium complexes as magnetic resonance imaging contrast agents (Review). Exp. Ther. Med..

[113] Jian W., Jia R., Wang J., Zhang H.-X., Bai F.-Q. (2019). Iron oxides with a reverse spinel structure: Impact of active sites on molecule adsorption. Inorg. Chem. Front..

[114] Muscas G., Yaacoub N., Concas G., Sayed F., Sayed Hassan R., Greneche J.M., Cannas C., Musinu A., Foglietti V., Casciardi S. (2015). Evolution of the magnetic structure with chemical composition in spinel iron oxide nanoparticles. Nanoscale.

[115] Ni D., Zhang J., Wang J., Hu P., Jin Y., Tang Z., Yao Z., Bu W., Shi J. (2017). Oxygen Vacancy Enables Markedly Enhanced Magnetic Resonance Imaging-Guided Photothermal Therapy of a Gd^3+^-Doped Contrast Agent. ACS Nano.

[116] Zuo X., Wang X., Si G., Zhang D., Yu X., Guo Z., Gu N. (2024). Size-Dependent Oxygen Vacancy of Iron Oxide Nanoparticles. Small Methods.

[117] Shao C., Shen A., Zhang M., Meng X., Song C., Liu Y., Gao X., Wang P., Bu W. (2018). Oxygen Vacancies Enhanced CeO_2_:Gd Nanoparticles for Sensing a Tumor Vascular Microenvironment by Magnetic Resonance Imaging. ACS Nano.

[118] Wu B., Deng K., Lu S.-T., Zhang C.-J., Ao Y.-W., Wang H., Mei H., Wang C.-X., Xu H., Hu B. (2021). Reduction-active Fe3O4-loaded micelles with aggregation- enhanced MRI contrast for differential diagnosis of Neroglioma. Biomaterials.

[119] Lu H., Chen A., Zhang X., Wei Z., Cao R., Zhu Y., Lu J., Wang Z., Tian L. (2022). A pH-responsive T1-T2 dual-modal MRI contrast agent for cancer imaging. Nat. Commun..

[120] Chen C., Ge J., Gao Y., Chen L., Cui J., Zeng J., Gao M. (2022). Ultrasmall superparamagnetic iron oxide nanoparticles: A next generation contrast agent for magnetic resonance imaging. WIREs Nanomed. Nanobiotechnol..

[121] Jin L., Yang C., Wang J., Li J., Xu N. (2022). Recent Advances in Nanotheranostic Agents for Tumor Microenvironment–Responsive Magnetic Resonance Imaging. Front. Pharmacol..

[122] Grzelczak M., Liz-Marzán L.M., Klajn R. (2019). Stimuli-responsive self-assembly of nanoparticles. Chem. Soc. Rev..

[123] Salaam J., Minoshima M., Kikuchi K. (2023). Recent Advances in Activatable 19F Magnetic Resonance Imaging Nano-Probes for the Detection of Biomarkers. Anal. Sens..

[124] Xiong H., Liu L., Wang Y., Jiang H., Wang X. (2022). Engineered Aptamer-Organic Amphiphile Self-Assemblies for Biomedical Applications: Progress and Challenges. Small.

[125] Gao Z., Hou Y., Zeng J., Chen L., Liu C., Yang W., Gao M. (2017). Tumor Microenvironment-Triggered Aggregation of Antiphagocytosis 99mTc-Labeled Fe_3_O_4_ Nanoprobes for Enhanced Tumor Imaging In Vivo. Adv. Mater..

[126] Lu J., Sun J., Li F., Wang J., Liu J., Kim D., Fan C., Hyeon T., Ling D. (2018). Highly Sensitive Diagnosis of Small Hepatocellular Carcinoma Using pH-Responsive Iron Oxide Nanocluster Assemblies. J. Am. Chem. Soc..

[127] Li F., Liang Z., Liu J., Sun J., Hu X., Zhao M., Liu J., Bai R., Kim D., Sun X. (2019). Dynamically Reversible Iron Oxide Nanoparticle Assemblies for Targeted Amplification of T1-Weighted Magnetic Resonance Imaging of Tumors. Nano Lett..

[128] Fu S., Cai Z., Ai H. (2021). Stimulus-Responsive Nanoparticle Magnetic Resonance Imaging Contrast Agents: Design Considerations and Applications. Adv. Healthc. Mater..

[129] Hou J., Liu H., Ma Q., Xu S., Wang L. (2022). Coordination-Driven Self-Assembly of Iron Oxide Nanoparticles for Tumor Microenvironment-Responsive Magnetic Resonance Imaging. Anal. Chem..

[130] Choi J.-s., Kim S., Yoo D., Shin T.-H., Kim H., Gomes M.D., Kim S.H., Pines A., Cheon J. (2017). Distance-dependent magnetic resonance tuning as a versatile MRI sensing platform for biological targets. Nat. Mater..

[131] Shin T.-H., Kang S., Park S., Choi J.-s., Kim P.K., Cheon J. (2018). A magnetic resonance tuning sensor for the MRI detection of biological targets. Nat. Protoc..

[132] Liu J., Yu W., Han M., Liu W., Zhang Z., Zhang K., Shi J. (2021). A specific “switch-on” type magnetic resonance nanoprobe with distance-dominate property for high-resolution imaging of tumors. Chem. Eng. J..

[133] Gao J., Wang Y., Meng X., Wang X., Han F., Xing H., Lv G., Zhang L., Wu S., Jiang X. (2024). A FAPα-activated MRI nanoprobe for precise grading diagnosis of clinical liver fibrosis. Nat. Commun..

[134] Wang C., Sun W., Zhang J., Zhang J., Guo Q., Zhou X., Fan D., Liu H., Qi M., Gao X. (2021). An electric-field-responsive paramagnetic contrast agent enhances the visualization of epileptic foci in mouse models of drug-resistant epilepsy. Nat. Biomed. Eng..

[135] Wang Z., Xue X., Lu H., He Y., Lu Z., Chen Z., Yuan Y., Tang N., Dreyer C.A., Quigley L. (2020). Two-way magnetic resonance tuning and enhanced subtraction imaging for non-invasive and quantitative biological imaging. Nat. Nanotechnol..

[136] Zhou Z., Huang D., Bao J., Chen Q., Liu G., Chen Z., Chen X., Gao J. (2012). A synergistically enhanced T(1)–T(2) dual-modal contrast agent. Adv. Mater..

[137] Geraldes C.F.G.C. (2024). Rational Design of Magnetic Nanoparticles as T1–T2 Dual-Mode MRI Contrast Agents. Molecules.

[138] Skotland T., Iversen T.-G., Sandvig K. (2010). New metal-based nanoparticles for intravenous use: Requirements for clinical success with focus on medical imaging. Nanomed. Nanotechnol. Biol. Med..

[139] Nowak-Jary J., Machnicka B. (2023). In vivo Biodistribution and Clearance of Magnetic Iron Oxide Nanoparticles for Medical Applications. Int. J. Nanomed..

[140] Kawai Y., Smedsrød B., Elvevold K., Wake K. (1998). Uptake of lithium carmine by sinusoidal endothelial and Kupffer cells of the rat liver: New insights into the classical vital staining and the reticulo-endothelial system. Cell Tissue Res..

[141] Murray P.J., Wynn T.A. (2011). Protective and pathogenic functions of macrophage subsets. Nat. Rev. Immunol..

[142] Couto D., Freitas M., Costa V.M., Chisté R.C., Almeida A., Lopez-Quintela M.A., Rivas J., Freitas P., Silva P., Carvalho F. (2016). Biodistribution of polyacrylic acid-coated iron oxide nanoparticles is associated with proinflammatory activation and liver toxicity. J. Appl. Toxicol..

[143] Alexis F., Pridgen E., Molnar L.K., Farokhzad O.C. (2008). Factors Affecting the Clearance and Biodistribution of Polymeric Nanoparticles. Mol. Pharm..

[144] Weissleder R., Stark D.D., Engelstad B.L., Bacon B.R., Compton C.C., White D.L., Jacobs P., Lewis J. (1989). Superparamagnetic iron oxide: Pharmacokinetics and toxicity. Am. J. Roentgenol..

[145] Feng Q., Liu Y., Huang J., Chen K., Huang J., Xiao K. (2018). Uptake, distribution, clearance, and toxicity of iron oxide nanoparticles with different sizes and coatings. Sci. Rep..

[146] Yang L., Kuang H., Zhang W., Aguilar Z.P., Xiong Y., Lai W., Xu H., Wei H. (2015). Size dependent biodistribution and toxicokinetics of iron oxide magnetic nanoparticles in mice. Nanoscale.

[147] Shao D., Lu M.-m., Zhao Y.-w., Zhang F., Tan Y.-f., Zheng X., Pan Y., Xiao X.-a., Wang Z., Dong W.-f. (2017). The shape effect of magnetic mesoporous silica nanoparticles on endocytosis, biocompatibility and biodistribution. Acta Biomater..

[148] Wei Y., Zhao M., Yang F., Mao Y., Xie H., Zhou Q. (2016). Iron overload by Superparamagnetic Iron Oxide Nanoparticles is a High Risk Factor in Cirrhosis by a Systems Toxicology Assessment. Sci. Rep..

[149] Arami H., Khandhar A., Liggitt D., Krishnan K.M. (2015). In vivo delivery, pharmacokinetics, biodistribution and toxicity of iron oxide nanoparticles. Chem. Soc. Rev..

